# Slowpoke: An Automated Golden Gate Cloning Workflow for Opentrons OT‑2 and Flex

**DOI:** 10.1021/acssynbio.5c00629

**Published:** 2026-02-05

**Authors:** Koray Malcı, Fankang Meng, Henri Galez, Alicia Franja Da Silva, Joaquin Caro-Astorga, Gregory Batt, Tom Ellis

**Affiliations:** † Department of Bioengineering, 4615 Imperial College London, London SW7 2AZ, U.K.; ‡ Imperial Centre for Engineering Biology, Imperial College London, London SW7 2AZ, U.K.; § Institut Pasteur, Inria, Université Paris Cité, 75015 Paris, France; ∥ 4914 London South Bank University, London SE1 0AA, U.K.; ⊥ LSBU Bioscience and Bioengineering Research Centre, London SE1 0AA, U.K.; ¶ IFP Energies Nouvelles, Rueil-Malmaison 92852, France

**Keywords:** automation, synthetic biology, Golden Gate cloning, high-throughput DNA assembly, standardization, genetic toolkits

## Abstract

In synthetic biology, DNA assembly is a routine process where increasing demands for standardization, high-throughput capacity, and error-free execution are driving the development of accessible, automated solutions. Here, we present Slowpoke, a user-friendly and flexible workflow for Golden Gate-based cloning designed for the popular entry-cost, open-source liquid-handling platforms Opentrons OT-2 and Flex. Slowpoke automates the key steps of the DNA assembly process, including cloning, *Escherichia coli* transformation, plating, and colony PCR, requiring user intervention primarily for colony picking and plate transfers. To further simplify the usage, we developed a free graphical user interface (GUI), available at https://slowpoke.streamlit.app/, which enables rapid protocol generation through simple file uploads. We validated the workflow using two Golden Gate-based toolkits, the MoClo Yeast Toolkit (YTK), and SubtiToolKit (STK). High assembly efficiencies were achieved across platforms for basic transcript unit constructions: 17/17 positive colonies with YTK on OT-2, 11/12 on Flex, and 8/13 with STK on OT-2. High-throughput assemblies were also performed with six parts in Flex using YTK-compatible parts, and 55 out of 57 combinations resulted in correct constructs. These results confirm the robustness and adaptability of the workflow across toolkit complexity and automation platforms. The Slowpoke suite, including code scripts and templates, is freely available at https://github.com/Tom-Ellis-Lab/Slowpoke, offering an accessible and modular solution for automating Golden Gate cloning in synthetic biology laboratories.

## Graphical abstract

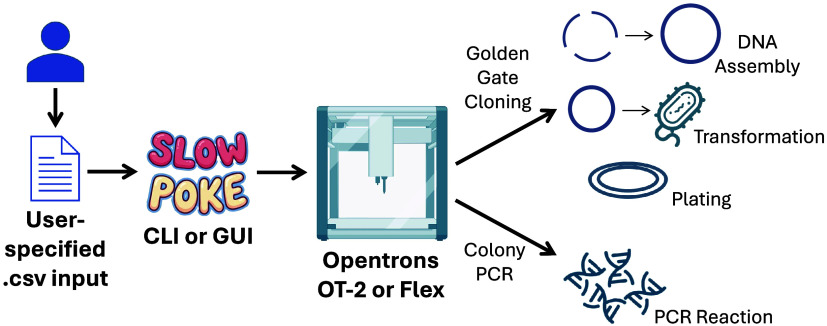

## 1 Introduction

Standardizing biological constructs and methodologies stands as a key objective within the field of synthetic biology.^1^ This involves the development of genetic engineering toolkits comprising well-characterized libraries of modular DNA parts encoding diverse functions^2^ and standardized assembly methods, such as Golden Gate Assembly,^3^ which use type II restriction enzymes to construct plasmids from parts in hierarchical order.^4^

Golden Gate Assembly-based genetic engineering toolkits have been developed for various organisms used in synthetic biology, including *Escherichia coli*,^5^ *Saccharomyces cerevisiae*,^6^ *Pichia pastoris* (*Komagataella phaffii*),^7^ *Bacillus subtilis*,^8^ *Komagataeibacter rhaeticus*,^9^ and more. Their standardization, versatility, robustness, and efficiency make them a routine part of work in many synthetic biology laboratories, as they also allow multiplexing and the construction of combinatorial DNA libraries.

Despite their advantages, the entire process of DNA assembly from parts library to transformation and colony screening can be laborious, time-consuming, and error-prone, especially for high-throughput studies.^10^ Consequently, there has been a focus on automation efforts using software, *in silico* pipelines, and liquid-handling platforms, especially at Biofoundry-scale.^11–13^ However, many automated workflows rely on expensive high-performance platforms out of reach for many laboratories. In contrast, the more accessible Opentrons liquid-handling robots, being cost-effective and open-source, have become commonplace at many institutions, particularly due to their use for high-throughput testing during the COVID-19 pandemic.^14^

Here, we develop an open-source automated plasmid construction program named Slowpoke, designed to be compatible with MoClo-format Golden Gate-based toolkits and the Opentrons OT-2 and Flex platforms. Slowpoke automates DNA assembly, chemical transformation, and plating in a single workflow and then uses colony PCR for the screening of the colonies in a second workflow. We validate Slowpoke for DNA cloning using the Yeast Toolkit (YTK) and SubtiToolKit (STK) and demonstrate it working on both the OT-2 and the newer Opentrons Flex system. Alongside this, we also describe an online tool with a graphical user interface (GUI) for Slowpoke Golden Gate cloning and colony PCR to further enhance accessibility. *Slowpoke* playfully references the iconic Pokémon character, whose approachable and relaxed nature reflects the tool’s design philosophy: to be user-friendly and to reduce the manual burden on researchers by providing a calm, guided, and reliable experience, so they can focus on the science.

## 2 Materials and Methods

### 2.1 Oligonucleotides, Reagents, and Plasmids

All primers used in the study are given in Table S1. The primers were ordered from Integrated DNA Technologies (IDT) as standard DNA oligos. Phire Plant Direct PCR Master Mix (Thermo Fisher Scientific) or DreamTaq Master Mix (Thermo Fisher) was used for colony PCR. Corresponding entry-level parts were chosen from MoClo Yeast Toolkit (YTK) or SubtiToolKit (STK). For level 1 assemblies using YTK, green fluorescent protein (GFP) in type 3 formation, was used, while a type 0C GFP was used for STK assemblies as a reporter. For high-throughput YTK assemblies, type-3b endolysin and single-chain variable fragment (scFv) parts were combined with type-3a signal peptides or type-4a tags, accordingly, a type-4b terminator was used. Golden Gate DNA assembly reactions were performed using Type IIS restriction enzyme, BsaI-HF, and T4 ligase or NEBridge Golden Gate Assembly Kit (BsaI-HF v2) from New England Biolabs (NEB). Assembled YTK plasmids were linearized using NotI-HF restriction enzyme (NEB) with rCutSmart buffer (NEB)

### 2.2 Strains and Growth Media

For bacterial transformation steps, either *E. coli* NEB Turbo Competent cells or NEB 5-α F’*Iq* Competent cells were used. Unless otherwise stated, all chemicals were sourced from Sigma-Aldrich. Standard cultivation involved growing the bacteria in 3 mL of LB media at 37 °C with shaking at 250 rpm for proper aeration. For selection media, the LB agar medium was supplemented with appropriate antibiotics (chloramphenicol, 34 μg/mL; ampicillin, 100 μg/mL; or kanamycin, 50 μg/mL). Similarly, *Bacillus subtilis* 168 strain was grown in LB at 37 °C with shaking at 250 rpm. For selection pressure for the transformants, LB medium containing erythromycin (5 μg/mL) was used.

*S. cerevisiae* strain, BY4741 {MATa; *his3*Δ1; *leu2*Δ0; *met15*Δ0; *ura3*Δ0}, was used for genomic integrations of the assembled YTK plasmids. For the cultivation of yeast strains, YPD medium containing yeast extract (1% (w/v)), peptone (2% (w/v)), and 2% (w/v) dextrose (glucose) was used. To select positive transformants expressing the *LEU2* marker, a synthetic defined medium containing a complete supplement mixture without leucine, 0.17% (w/v) yeast nitrogen base without amino acids, 0.5% (w/v) ammonium sulfate, 2% (w/v) glucose, and 2% (w/v) agar was used.

### 2.3 Slowpoke Protocol

Python scripts were developed using Spyder 5.4 and are compatible with the Opentrons API 2.0 for OT-2 and 2.21 for the Flex. The scripts use standard Python libraries (tkinter, csv, json, os, and sys) to guide the user through file selection and automatically generate plate maps and an Opentrons-compatible protocol based on input combinations. The script produces a finalized CSV layout and appends part combinations to a template workflow file for downstream robotic execution. No external packages or APIs were required. The codes are available at (https://github.com/Tom-Ellis-Lab/Slowpoke). The online graphical user interface was developed by using the Streamlit Python framework.

### 2.4 Automated Golden Gate Reaction

For the Opentrons OT-2 associated workflows, a one-tube Golden Gate reaction was set in 10 μL of reaction volume containing 50 fmol of the entry-level plasmids and the backbone plasmids with 0.5 μL (10 units) of BsaI and 0.5 μL (200 units) of T4 ligase in 1× T4 ligase buffer. A master mix was first prepared depending on the total reaction number, and then it was distributed to the single tubes without custom DNA parts. For the Opentrons Flex workflow, a one-tube Golden Gate reaction was set in 12 μL of reaction volume containing 25 fmol of the entry-level plasmids and the backbone plasmids with 1.2 μL of Golden Gate Enzyme Mix in 1× T4 ligase buffer. Ligase buffer and water were first dispensed in the reaction well, followed by the plasmids and the enzymes. The reaction mixture was cycled in either the Opentrons thermocycler unit or the standard benchtop thermocycler 25 times at 37 °C for 2 min and 16 °C for 5 min. The reaction mixture was then incubated at 60 °C for 5 min to denature the enzymes and was directly used to transform *E. coli* and plated on 6-well plates with 5 mL LB agar plates with the corresponding antibiotics

### 2.5 Automated Colony PCR

A master mix was first prepared containing water, colony PCR primers, and 2× PCR master mix, either Phire Plant Direct (Thermo Fisher) for the OT-2 workflow or DreamTaq (Thermo Fisher) for the Flex workflow. Then, 9 μL of this master mix was dispensed into each reaction tube, followed by the addition of 1 μL from the colony template plates for the OT-2 platform. The master mix volume was set to 13 μL, and 2 μL from colony templates were used for the Flex platform. PCR reactions were set up according to the manufacturer’s protocol, with adjustments made to extension times and annealing temperatures based on fragment size and primers. The OT-2 workflow employed the Opentrons thermocycler module (with additional benchtop thermocyclers as needed), while the Flex workflow used benchtop thermocyclers

Details of the reaction volumes for both the Golden Gate and colony PCR protocols on the OT-2 and Flex platforms are provided in Table S5.

### 2.6 Yeast Transformation

Yeast transformations were performed using the lithium acetate (LiOAc) method. Strains were grown overnight in YPD at 30 °C, diluted 1:100 into 15 mL of fresh YPD, and incubated at 30 °C for 4–6 h until OD_600_ reached 0.8–1.0. Cells were harvested, washed with 0.1 M LiOAc, and resuspended in the same buffer to yield 100 μL per transformation. For each reaction, 100 μL of cells was pelleted and resuspended in 64 μL of DNA mix containing 500 ng of linearized DNA, 10 μL boiled salmon sperm DNA (Invitrogen), and sterile water. Then, 296 μL of PEG/LiOAc (260 μL of 50% PEG-3350 (Sigma-Aldrich) + 36 μL of 1 M LiOAc) was added and mixed thoroughly. The mixture was heat shocked at 42 °C for 40 min, pelleted, and resuspended in 200 μL of 5 mM CaCl_2_. Cells were plated onto selective dropout plates and incubated at 30 °C for 2–4 days until colonies appeared.

### 2.7 *Bacillus subtilis* Transformation

To transform *Bacillus subtilis* 168, a single colony was picked from a fresh plate and used to inoculate 3 mL of MC medium (Table S2). The culture was incubated at 37 °C with shaking until it reached an OD_600_ between 1.1 and 1.5, corresponding to the final logarithmic or early stationary growth phase (approximately 4–5 h). For transformation, 1 mL of this culture was mixed with 2–3 μL of plasmid DNA (containing 0.5–2 μg of DNA) in a sterile 1.5 mL microcentrifuge tube. The mixture was vortexed briefly for 5 s and incubated for 40 min at 37 °C with shaking. The culture was centrifuged, and the pellet was resuspended in 75 μL of medium and was plated onto selective LB agar. Plates were incubated overnight at 37 °C. The following day, individual colonies were picked and cultured in 3 mL of selective LB liquid medium containing 5 μg/mL erythromycin with shaking at 250 rpm, for further analysis.

### 2.8 Flow Cytometry Analyses

Cultures were diluted 1/100 into phosphate-buffered saline (PBS) to a final volume of 200 μL in a 96-well plate. Measurements were taken by using an Attune NxT Acoustic Focusing Cytometer with an autosampler module. For *B. subtilis* readings, voltage settings were 440 V for FSC, 340 V for SSC, and 490 V for BL1 to measure GFPmut3b expression. For *S. cerevisiae*, FSC 300 V, SSC 350 V, and BL1 500 for sfGFP were used. A total of 10,000 events were recorded per sample. Using a previously described gating approach,^6^ singlets were gated using FSC-H × FSC-A. Data from the flow cytometer were analyzed and visualized using FlowJo 10.10.0. Samples were collected at 6 h of cultivation, representing the midexponential phase. For characterizing inducible promoters, inducers were added when the cultures reached the early stationary phase (approximately 2 h after inoculation). The experiments were conducted in triplicate.

## 3 Results

### 3.1 Slowpoke Overview

We developed two complementary workflows, one for Golden Gate cloning and one for colony PCR, that are designed to be user-friendly and run entirely offline via the generator.py script in the command line. To further improve accessibility, we also developed an online version of Slowpoke with an intuitive, user-friendly interface. In both cases, users simply provide the required input files, and the program generates the protocol with a single click. The offline standalone tool and the web-based GUI guide users step-by-step through protocol design and execution. Protocol generation relies on user-supplied.csv files that define the layout of genetic parts and reagents. For Golden Gate assembly in the cloning workflow, users provide three.csv files: a *fixed toolkit map*, a *custom parts map*, and a *combination file* specifying how the parts are assembled. The fixed toolkit map corresponds to a standardized genetic toolkit (for example, a MoClo or YTK set obtained from Addgene) typically stored in a 96-well plate layout. In practice, many laboratories replicate these Addgene plates in the same format as that of a working plasmid source. The custom parts map contains user-designed parts that are compatible with the corresponding toolkit and are intended for specific applications. While this two-plate layout simplifies combinations, users may freely select and combine parts from either or both plates when generating protocols. Figure 1 illustrates the general workflows of both programs.

**Figure 1:**
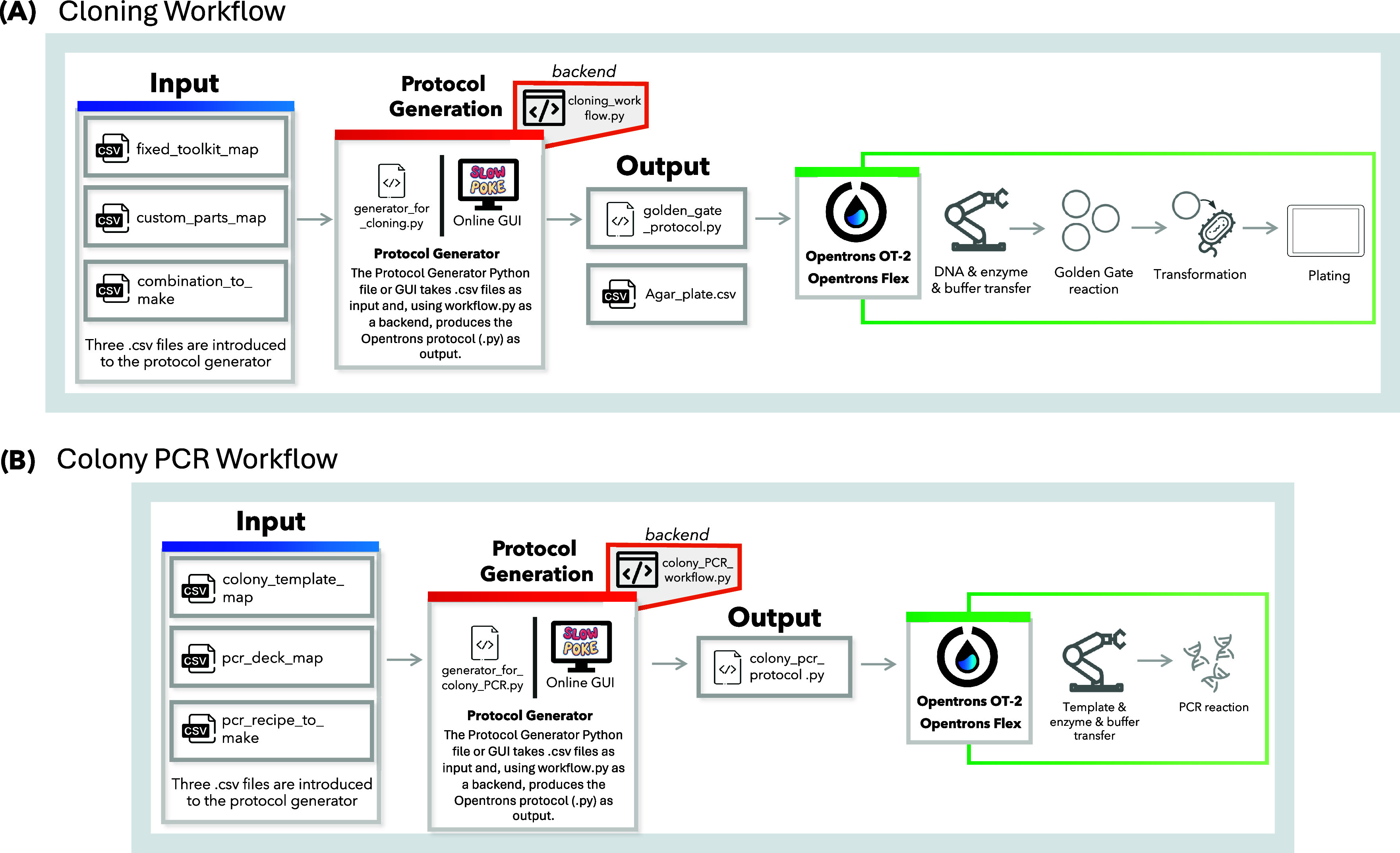
Schematic overview of the protocol design workflows developed for the Opentrons platform. Protocols can be generated using either the generator.py Python script via the command line or the online Slowpoke tool, which features a user-friendly GUI. Both tools run the workflow.py files in the backend. (A) Workflow for Golden Gate-based cloning, where users define genetic part layouts and assembly combinations. (B) Workflow for colony PCR, including colony selection, reagent layout, and reaction recipe input.

For the cloning workflow, users specify the arrangement of DNA parts and assembly combinations. For the colony PCR workflow, users input the positions of the picked colonies, define the layout of PCR reagents in the tube holder, and specify the reaction mix recipes. Based on this information, the protocol generators automatically compile executable scripts for the Opentrons robots using the workflow.py files in the backend. The resulting protocols can be directly uploaded to the Opentrons app and executed on the robot without further modification. Detailed guidance is provided in the Supporting Information under the “Guidance On Slowpoke” section.

It should be noted that certain user-specific parameters, such as restriction enzyme–specific temperatures and incubation times for Golden Gate assembly or DNA polymerase–specific settings for colony PCR, may need to be adjusted in the workflows. These modifications can be made easily by editing the relevant code in any text editor, as described in detail in the Supporting Information and the online README file.

Slowpoke supports the simultaneous preparation of up to 96 Golden Gate assemblies and subsequent colony PCR reactions in the semiautomated workflow. In a standard Golden Gate cloning protocol using the Opentrons OT-2, a single thermocycler module handles both the assembly reactions and the heat-shock transformation of *E. coli*. However, since the thermocycler occupies four out of 11 available deck slots, the remaining space must be allocated carefully, usually to a temperature module, tip racks, source plates containing DNA parts, and one or two agar plates.

To optimize the deck space and increase flexibility, the workflow can be modified to replace the thermocycler with standard benchtop thermocyclers. This adjustment allows the protocol to run on just the basic OT-2 unit equipped with a temperature module, freeing up the deck slots and expanding the capacity. For the colony PCR step, the deck layout includes a tube rack for reagents, a source plate for colonies, and a PCR mix plate for dispensing the master mix. The remaining slots can be allocated to 96-well PCR plates containing the reaction mixtures.

When additional external thermocyclers are available, the workflow can be scaled further. For example, we have successfully performed 288 colony PCR reactions (three full 96-well plates) in parallel by preparing the reactions using OT-2 and running them using two additional benchtop thermocyclers. Gel electrophoresis results of 236 out of 288 reactions to screen the transformants for juxtracrine signaling plasmids^15^ are shown in Table S4 and Figure S4.

### 3.2 Validation of Slowpoke for Yeast Synthetic Biology

To validate the Slowpoke workflow on the OT-2 platform, we first looked at the automation of plasmid assembly for engineering expression in yeast. We used Slowpoke to automatically assemble Level 1 transcription units (TUs) using 19 different promoters from the MoClo Yeast Toolkit (YTK), with a yeast-optimized superfolder green fluorescent protein (sfGFP) in part 3 format as the reporter (Figure 2A). Each TU was assembled into a backbone vector plasmid (pWS064) that includes a LEU2 selection marker, homology arms for genomic integration, and an *E. coli* GFP dropout cassette for visual selection of correct assemblies (Figure 2B).

**Figure 2:**
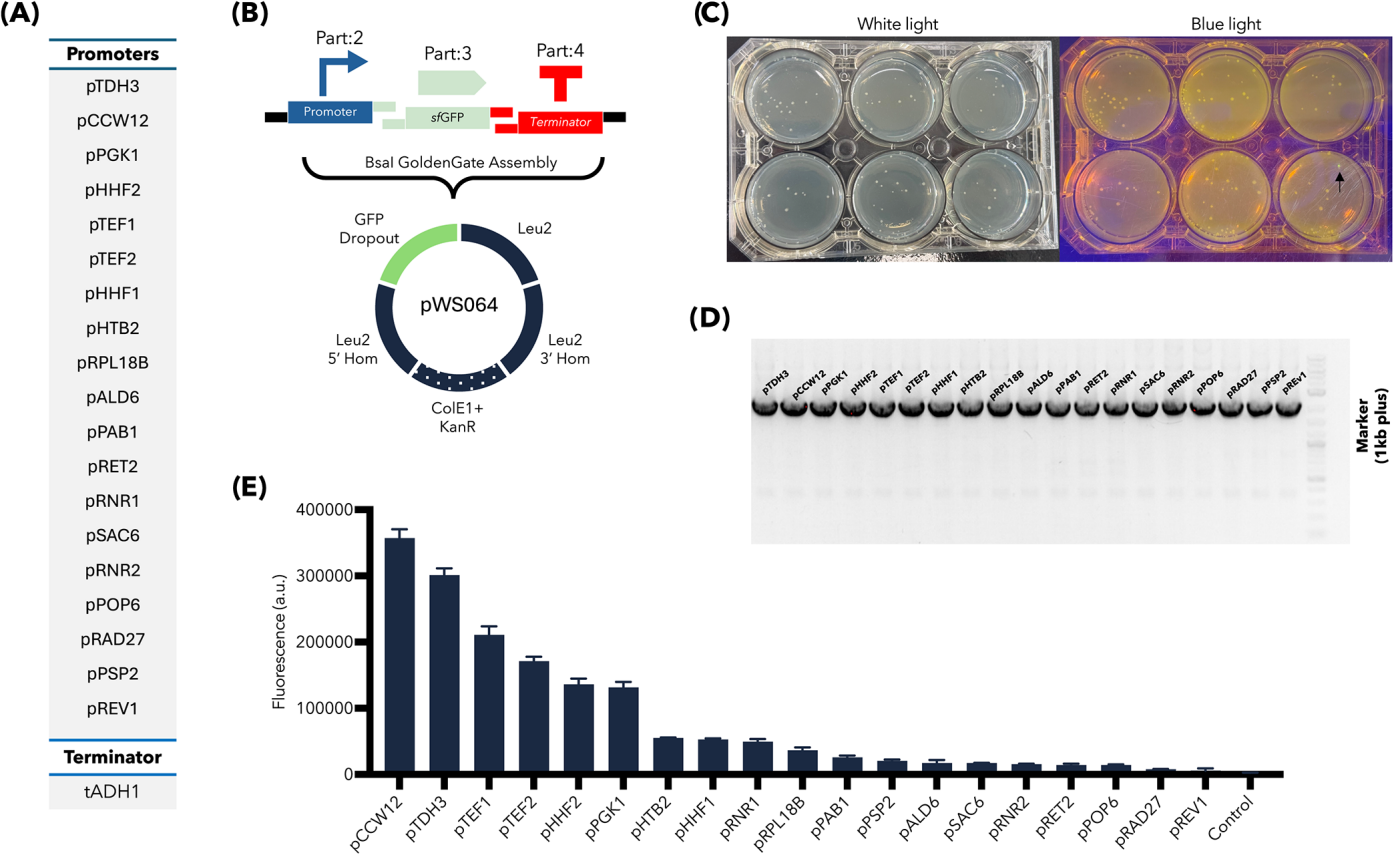
Validation of Slowpoke performance for YTK-compatible plasmid assemblies on the OT-2 platform. (A) Promoters and terminators used in the construction of Level 1 transcription units (TUs). (B) Schematic overview of the Golden Gate assembly using selected genetic parts and the pWS064 backbone plasmid. *E. coli*-specific elements (origin of replication and selection marker) are indicated with dotted parts. (C) Representative 6-well plate showing successfully transformed white colonies under daylight and blue light illumination. Green fluorescent colonies are marked with black arrows. (D) Colony PCR result of the randomly selected 19 colonies. (E) sfGFP expression levels in *S. cerevisiae* transformed with the assembled plasmids, highlighting differences in promoter strength. Error bars represent the standard deviations of three replicates. It should be noted that the *E. coli* GFP dropout cassette in pWS064 facilitates selection: green fluorescent colonies indicate an intact vector (false positive), while nonfluorescent (white) colonies suggest successful insertion. The sfGFP gene of interest is expressed only in yeast under yeast promoter control.

The OT-2 successfully plated the *E. coli* transformation mixtures by dispensing 4.5 μL droplets (this was set to 2.5 μL for Flex) of each transformation reaction onto the surface of a 5 mL of kanamycin-supplemented LB agar in a 6-well plate. Dispensing was performed near the agar surface using a calibrated drop-height (*z* ≈ 5 mm), which avoids contact with the agar while ensuring accurate placement. Thirteen unique *x*–*y* placement positions on the agar plate were defined for plating. These parameters can be adjusted by users for different agar thicknesses or labware. As shown in a representative plate (Figure 2C), the majority of *E. coli* colonies were not fluorescent, indicating they were candidates for correct assemblies since the yeast promoters are inactive in *E. coli*, preventing GFP expression from the reporter CDS (pWS033). Only a single green fluorescent colony carrying an intact pWS064 plasmid was observed (black arrow in Figure 2C).

We then performed colony PCR on 19 randomly selected white colonies (one colony from each construct). All 19 PCR products yielded the expected size for a correct assembly (Figure 2D), demonstrating a high assembly accuracy. To verify the functionality of the assembled constructs in the target host, we extracted plasmids from these colonies and transformed them into *S. cerevisiae*. Flow cytometry was used to quantify the sfGFP expression from the resulting engineered yeast (Figure 2E).

As expected and consistent with the original MoClo YTK characterization,^16^ use of strong promoters such as pTDH3 and pCCW12 resulted in high levels of GFP fluorescence in the engineered yeast, whereas weak promoters like pREV1 and pRAD27 produced lower signals. These experimental results confirm that the Slowpoke workflow can reliably assemble Golden Gate-compatible parts and that colony PCR is an effective predictor of correct assemblies suitable for transformation into the final host organism.

### 3.3 Slowpoke Validated with a Second Toolkit

To demonstrate the versatility of the Slowpoke workflow across different Golden Gate-compatible toolkits, we assembled five-part Level 1 TUs using a Gram-positive bacterial toolkit, SubtiToolKit (STK). These assemblies incorporated ribosome binding site (RBS) parts alongside one constitutive and one mannitol-inducible promoter with varying RBS strengths (Figure 3A). The TUs were cloned into the *Bacillus subtilis* expression vector STK202, generating three distinct GFP-expressing constructs (Figure 3B,C). STK202 contained a LacZ dropout cassette for blue–white screening of transformants since a GFP dropout cassette could interfere with the *B. subtilis* GFP-expressing construct in *E. coli*.

**Figure 3:**
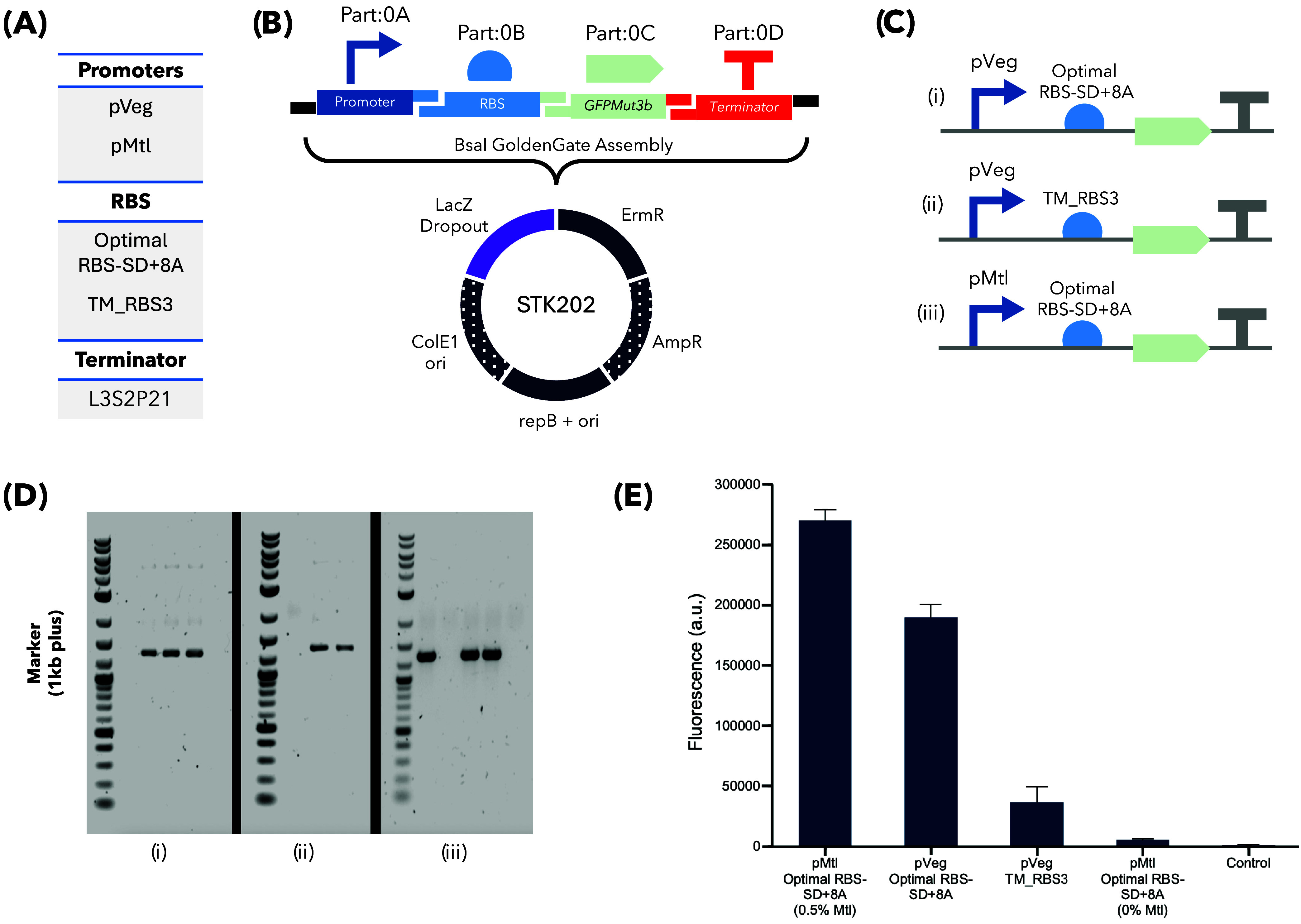
Validation of Slowpoke performance for STK-compatible plasmid assemblies on the OT-2 platform. (A) Promoters, RBSs, and terminator used in the construction of Level 1 TUs. (B) Schematic of the Golden Gate Assembly showing the selected genetic parts and the STK202 backbone plasmid. *E. coli*-specific elements (origin of replication and selection marker) are indicated with dotted parts. (C) SBOL diagrams of the assembled TUs, showing combinations of constitutive or mannitol-inducible promoters with strong or midweak RBSs. (D) Colony PCR result of the randomly selected 13 colonies from three constructs. (E) sfGFP expression levels in *B. subtilis* transformed with the assembled plasmids, highlighting differences in promoter strength. Error bars represent the standard deviations of three replicates.

Transformation plates yielded a high proportion of white colonies, with blue colonies representing less than 5% of the total, which was comparable to manual workflows.^8^ Colony PCR screening of randomly selected white colonies, five from constructs I and III, and three from construct II, revealed that over 60% of colonies contained the expected amplicons (Figure 3D). Although this assembly efficiency was lower than that observed with YTK assemblies, it remained comparable to that of manual protocols. The reduced efficiency is likely due to the increased number of parts and the use of *E. coli* as an intermediate host, which may negatively impact assembly yield when employing *E. coli*-active parts from *B. subtilis*. Nevertheless, the benefits of automation, such as higher throughput, standardization, and reduced human error, make this a reasonable trade-off for more complex assemblies.

To confirm the plasmid functionality, positive transformants were introduced into *B. subtilis*, and GFP expression was assayed. Consistent with the original STK characterization,^8^ strong GFP fluorescence was observed upon induction with 0.5% mannitol when the mannitol-inducible promoter was paired with a strong RBS (RBS-SD Optimal+8A). Constitutive promoter constructs also displayed expected expression levels corresponding to the RBS strength (Figure 3E). GFP expression patterns were also consistent in the flow cytometry data (Figure S1).

These results illustrate that the Slowpoke workflow is compatible with multiple MoClo/Golden Gate toolkits. While automated assembly yields may decrease as part complexity increases, especially when *E. coli*-active parts are involved, the overall performance remains comparable to manual methods.

### 3.4 YTK Assemblies on the Flex Platform

To assess the compatibility of Slowpoke with a newer automation platform, we adapted our workflow to Opentrons Flex. Compared to the OT-2, Flex provides a larger deck capacity, integrated plate handling, improved pipetting accuracy, and a built-in touchscreen interface, eliminating the need for an external computer.

With only minor modifications, the original OT-2 protocols were adapted for use with Flex without a thermocycler module. All thermocycling steps were performed using standard laboratory PCR machines, while the remaining workflow steps remained largely unchanged.

To validate the adapted protocol, we repeated the assembly of sfGFP transcription units (TUs) using six different promoters from YTK, as illustrated in Figure 4. Transformation plates showed a high proportion of white colonies. Two colonies from each construct were randomly selected for colony PCR screening; 11 out of 12 colonies yielded the expected amplicons, demonstrating high assembly efficiency.

**Figure 4:**
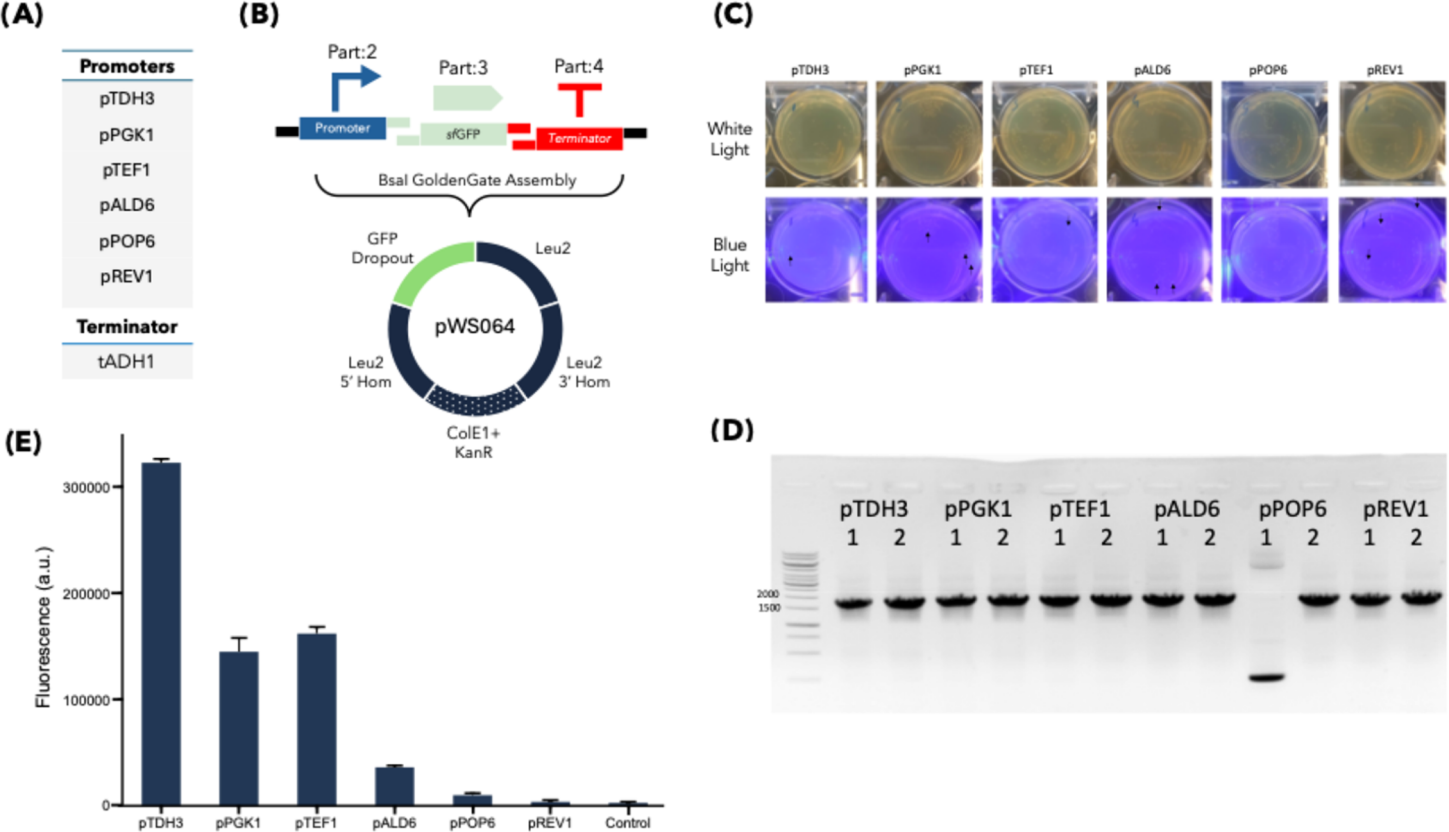
Adapting Slowpoke to the Opentrons Flex platform with a functional assay. (A) Promoters and the terminator are used in the construction of Level 1 TUs. (B) Schematic overview of the Golden Gate assembly using selected genetic parts and the pWS064 backbone plasmid. *E. coli*-specific elements (origin of replication and selection marker) are indicated with dotted parts. (C) Representative 6-well plate showing successfully transformed white colonies under daylight and blue light illumination. Green fluorescent colonies are marked with black arrows. (D) Colony PCR result of the randomly selected 12 colonies (two colonies from each construct). (E) sfGFP expression levels in *S. cerevisiae* transformed with the assembled plasmids, highlighting differences in promoter strength. Error bars represent the standard deviations of three replicates. It should be noted that the *E. coli* GFP dropout cassette in pWS064 facilitates selection: green fluorescent colonies indicate an intact vector (false positive), while nonfluorescent (white) colonies suggest successful insertion. The sfGFP Gene of Interest is expressed only in yeast under yeast promoter control.

To verify plasmid functionality, the assembled constructs were transformed into *S. cerevisiae*, and GFP expression was analyzed by flow cytometry. The resulting expression patterns were consistent with promoter strength,^16^ as expected. The distribution of GFP-expressing cells is also shown in Figure S2.

These results confirm that the Slowpoke workflow can be reliably implemented on the Opentrons Flex platform, achieving assembly efficiencies comparable to those obtained with the OT-2.

### 3.5 High-Throughput Validation on the Flex Platform

To evaluate the scalability of the Slowpoke workflow, we attempted the construction of 62 plasmids, each consisting of a 6-part YTK assembly, corresponding to more than 5000 robot-executed instructions. These constructs encoded secreted recombinant proteins, including an endolysin (a potential antimicrobial agent^17^) and a single-chain variable fragment (scFv),^18,19^ expressed from eight constitutive or light-inducible promoters, ten signal peptides, and two C-terminal tags (Figure 5A,B and Table S2).

**Figure 5:**
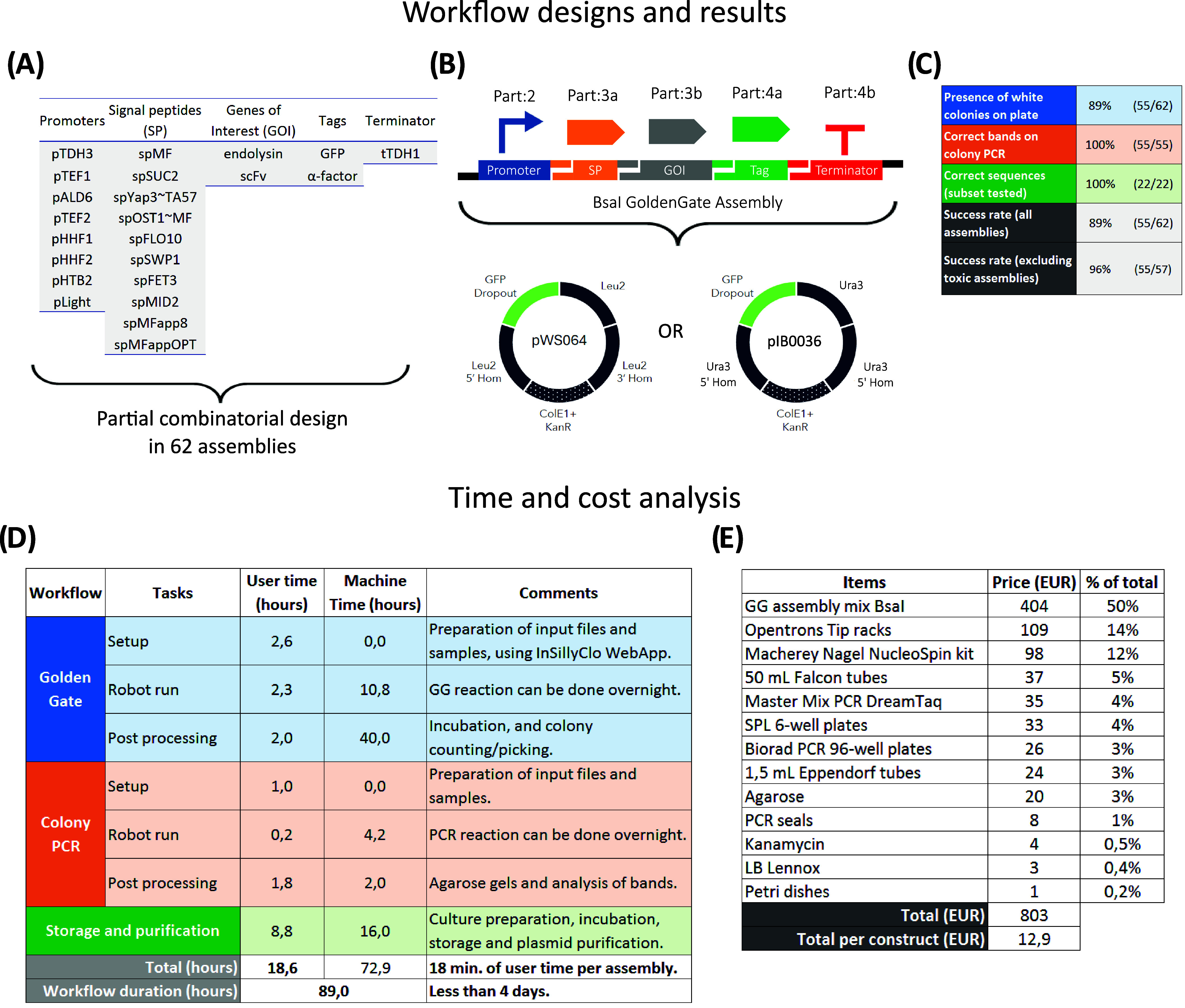
Large-scale cloning with the Opentrons Flex platform. (A) Genetic parts used in the construction of Level 1 TUs. (B) Schematic overview of the Golden Gate assembly using selected genetic parts and the backbone plasmids. *E. coli*-specific elements (origin of replication and selection marker) are indicated with dotted parts. (C) Success rate of each step of the workflow. One colony per assembly was tested by PCR, and a subset of 22 plasmids was checked by Sanger sequencing. (D) Breakdown of the workflow in tasks with corresponding user time and machine time (Flex robot, thermocycler, and incubator). (E) Costs of reagents for the realization of this 62-assembly workflow.

We obtained white colonies for 55 of the 62 assemblies, and all 55 isolates tested by colony PCR showed the expected band sizes (Figures 5C and S3). A subset of 22 plasmids was also verified by Sanger sequencing, confirming the correct assembly of the backbone, promoter, signal peptide, and gene of interest. All seven failed assemblies contained the endolysin CDS. These were repeated in a second run manually; two failed again, while three grew abnormally slowly and failed to grow in liquid culture. When excluding cases where toxicity is likely to be present (five assemblies), the Slowpoke workflow succeeded in 55 assemblies out of 57 assemblies (96%) as shown in Figure 5C. This high assembly efficiency, even with a relatively large number of parts, shows that Slowpoke can reliably automate plasmid construction when there are no other biological constraints, such as toxicity.

Slowpoke follows the same protocol across different-level assemblies in the toolkits; therefore, these results provide strong evidence that Slowpoke can also scale effectively to multigene or operon-level assemblies with corresponding Type IIS enzymes, which are essential for metabolic engineering workflows.

We also quantified user and machine time for this workflow (Figure 5D and Table S3). The entire process required less than 4 days, indicating that the workflow could be run within a typical working week. Of the 18.6 h of user time, approximately half was associated with strain storage and plasmid purification, which were performed manually but could be automated in 96-well format. The InSillyClo web application was used to automate plasmid map construction, PCR simulation, and the generation of the combinations_to_make.csv file, reducing setup and postprocessing time.^20^ Estimated reagent costs for this workflow are shown in Figure 5E, with Golden Gate enzymes, Opentrons tips, and plasmid purification kits representing 76% of the total cost.

This high-throughput demonstration using six YTK-compatible parts per assembly and yielding a high correct-assembly rate illustrates the robustness of Slowpoke for complex DNA assembly workflows. Therefore, Slowpoke not only accelerates strain construction but also minimizes human error in large combinatorial cloning tasks.

### 3.6 A Web-Based Graphical User Interface for Slowpoke

To further streamline protocol design and increase accessibility, we developed a free, browser-based graphical user interface (GUI) for the Slowpoke cloning workflow. The online tool is publicly available and allows users to generate automated cloning protocols with a simple, intuitive interface (Figure 6).

**Figure 6:**
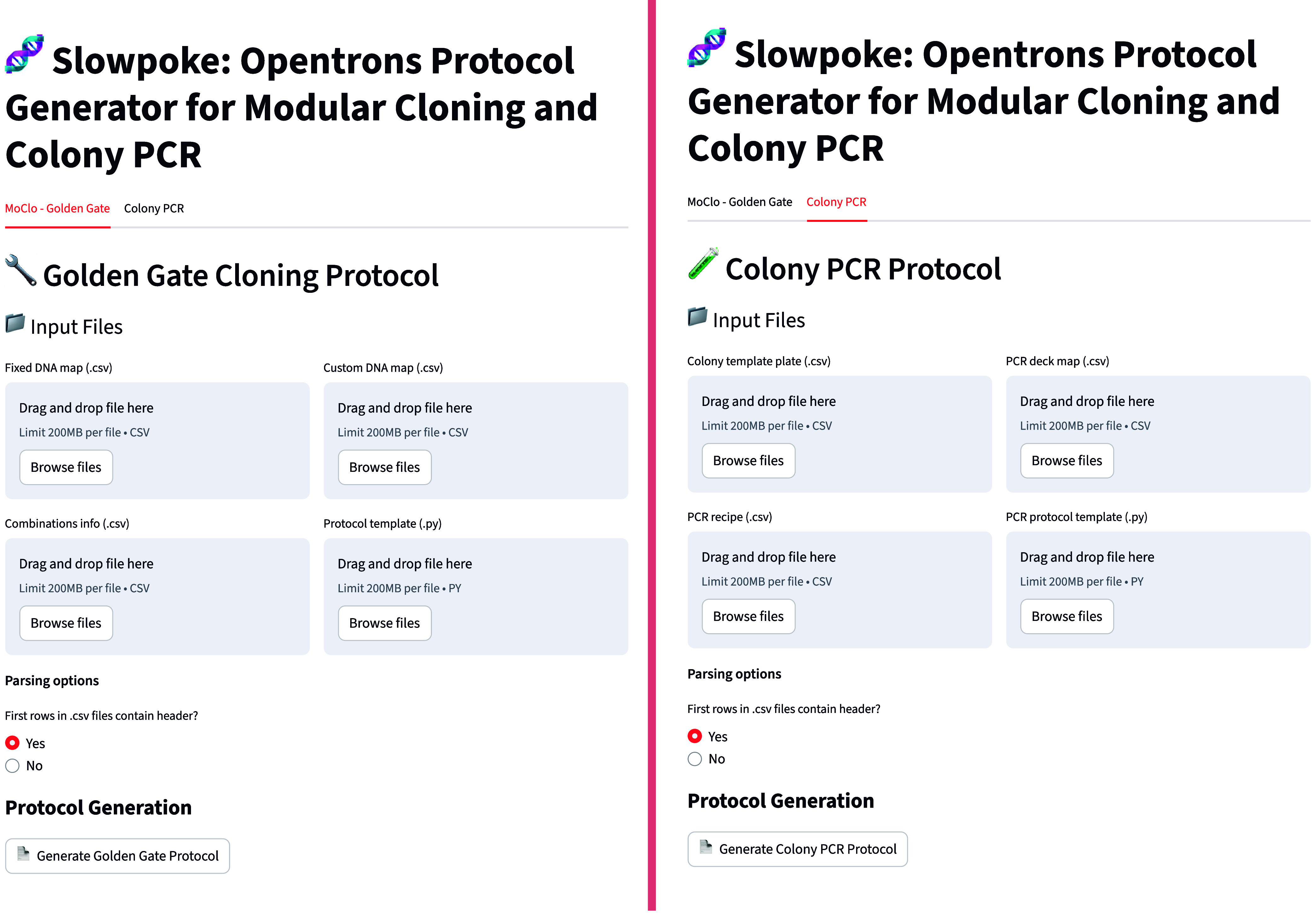
Graphical user interface (GUI) of the Slowpoke cloning workflow. The online interface is freely available to all users and enables rapid generation of Opentrons-compatible Golden Gate cloning protocols. Users can download and complete.csv template files for DNA part maps and assembly combinations, then upload them to the tool to automatically generate the robotic protocols.

To support ease of use, the GUI provides downloadable.csv templates with predefined structures as well as a detailed ReadMe file with visual guidance on how to complete each file. These templates minimize user input by specifying only the fields required for the generation of valid assembly combinations. This structured approach simplifies protocol setup and also provides a record of the selected parts and construct designs, which can be useful for reproducibility and future reference.

After the templates are completed with their specific parts and constructs, the files can be uploaded directly to the tool. With a single click, the tool executes the underlying protocol generation and workflow scripts, producing a ready-to-run Opentrons protocol.

We aim to maintain the online interface with regular updates that reflect new features and platform capabilities. In addition, the entire tool is open-source, allowing users to modify or extend the code to suit alternative use cases or develop new versions of the Slowpoke GUI.

## 4 Discussion

Using Slowpoke, we achieved high assembly efficiencies, over 90% with YTK and 60% with STK, consistent with values reported for manual Golden Gate assemblies using these toolkits.^6,8,16^ The assembled constructs were validated in*S. cerevisiae* and*B. subtilis*, confirming their robustness and functionality. Together, these results demonstrate that Slowpoke is a reliable and accessible automation tool for Golden Gate-based cloning with modularity and platform compatibility required for high-throughput synthetic biology applications.

In recent years, significant research efforts have focused on accelerating DNA assembly, particularly for large libraries and high-throughput applications. However, existing tools often present limitations: many require at least basic coding skills; others depend on advanced biofoundry-level infrastructure; and some are built on cloning methods that have not been widely adopted by the synthetic biology community.

As summarized in Table 1, Slowpoke places in a distinct position among existing DNA-assembly automation tools. AssemblyTron provides flexible Golden Gate and *in vivo* assembly automation on the OT-2, but it is a code-intensive Python package that is not compatible with Flex in its original implementation, making it less accessible to users without scripting experience. DNA-BOT enables automation of the BASIC method^27^ on the OT-2 and has demonstrated high throughput; however, BASIC is less widely adopted than MoClo/Golden Gate in many synthetic biology communities. DNAda and PlasmidMaker were developed for advanced biofoundry infrastructures and rely on sophisticated liquid-handling systems. Also, PlasmidMaker employs non-Type IIS PfAgo-based assembly chemistry. Therefore, they have different scopes and facility requirements than low-cost platforms. RoboMoClo targets multilevel MoClo workflows on integrated robotics systems but depends on specialized hardware. Slowpoke fills a gap by offering low-entry-cost, MoClo-compatible, end-to-end automation that spans Golden Gate assembly, transformation, plating, and colony PCR on both the OT-2 and Flex platforms while also providing a user-friendly, no-code graphical interface.

**Table 1:** Comparison of Slowpoke to Similar Studies

study	platform (robot types)	cloning method(s)	workflow coverage	user accessibility/interface type
Slowpoke (this study)	Opentrons OT-2 & Flex	MoClo/Golden-Gate (GG)	GG assembly → transformation → plating → colony PCR (manual picking)	online GUI; offline stand-alone application
AssemblyTron[Bibr ref21] (2022)	Opentrons OT-2	MoClo/Golden-Gate & homology-dependent in vivo assemblies	DNA assembly automation	Python package
DNA-BOT[Bibr ref11] (2020)	Opentrons OT-2	BASIC assembly	BASIC assembly → transformation → plating	offline stand-alone application
DNAda[Bibr ref12] (2023)	advanced robotic set-up/biofoundry	J5-directed assemblies^a^	design → worklist → plasmid construction → sample tracking (NGS)	online GUI; command-line Interface
PlasmidMaker[Bibr ref13] (2022)	advanced robotic set-up/biofoundry	PfAgo-based assembly	build (PCR, cleavage, ligation) → transformation → test → stock	online GUI; command-line/local code
RoboMoClo[Bibr ref22] (2022)	advanced robotic set-up/biofoundry	MoClo/Golden-Gate	multilevel GG assembly	hardware-integrated automation platform

a J5 ^23^ is a DNA assembly software supporting various cloning methods, including USER,^24^ Gibson^25^ CPEC,^26^ and Golden Gate.

Beyond Golden Gate assembly, Slowpoke can be extended to support other modular genetic toolkits that rely on alternative DNA assembly methods, such as Gibson Assembly or *in vivo* recombination.^28–30^ As long as the toolkit follows a standardized assembly scheme with interchangeable parts, it can be readily integrated into the workflow. Furthermore, a complementary open-source tool, named InSillyClo,^20^ can expand its design capabilities by generating large-scale cloning maps that integrate seamlessly with Slowpoke’s Opentrons protocols as demonstrated in the present study for the high-throughput assemblies on the Flex platform.

Despite these advantages, Slowpoke is not without limitations. Certain steps still require user intervention, such as sealing PCR plates in the OT-2 thermocycler module or transferring PCR tubes to a benchtop thermocycler in the Flex workflow. The most labor-intensive task remains colony picking, which Opentrons platforms currently lack support for. However, open-source solutions have emerged; for example, the Marburg iGEM 2019 team developed an open-source colony picker by equipping the Opentrons OT-2 with a 3D-printed camera and light table. The team used neural networks to detect colonies and guide robotic picking.^31^ Subsequently, a group from Imperial College London used this hardware concept but replaced the software with a modular system of a PiCam server, a Man-in-the-Middle API for colony detection, and an OT-2 Jupyter client, making the workflow more flexible and accessible.^32^ If such advances are integrated with Slowpoke, they could enable a fully automated, end-to-end cloning and verification pipeline with minimal human intervention.

## 5 Conclusion

DNA assembly is a fundamental task in synthetic biology, and the growing demand for higher throughput highlights the need for accessible automation. Slowpoke addresses this by providing a user-friendly, open-source workflow for Golden Gate-based cloning on low-cost Opentrons platforms, integrating DNA assembly, *E. coli* transformation, plating, and colony PCR. By automating these repetitive steps, Slowpoke minimizes human error and streamlines high-throughput experimentation.

We validated Slowpoke with both YTK and STK toolkits on Opentrons OT-2 and adapted it to the newer Flex platform, confirming its versatility across affordable, widely available automation systems. To further enhance accessibility, Slowpoke’s GUI is freely available at https://slowpoke.streamlit.app/.

With its open-source design, cross-platform compatibility, and ease of use, Slowpoke lowers barriers to laboratory automation and offers a practical solution for synthetic biology groups seeking to scale up and accelerate DNA assembly.

## Supplementary Material



## Data Availability

The codes can be found at https://github.com/Tom-Ellis-Lab/Slowpoke.

## Notes

Primers used in the study (Table S1); high-throughput DNA assembly combinations (Table S2); details of user and machine time for high-throughput DNA assembly (Table S3); high-throughput colony PCR details (Table S4); details of the reaction volumes (Table S5); MC medium components (Table S6); GFP expression patterns (Figures S1 and S2); colony PCR results for the large-scale assemblies (Figure S3); high-throughput colony results (Figure S4); the Opentrons Deck Layouts (Figures S5–S8); the GUI (Figure S9); headers in the input files (Figure S10). (PDF)
