# Supplementary material for: Slowpoke: An Automated Golden Gate Cloning Workflow for Opentrons OT‑2 and Flex
Source: ACS Synth Biol. 2026 Feb 5;15(2):511–21. doi: 10.1021/acssynbio.5c00629 (PMC12930513; doi:10.1021/acssynbio.5c00629)
Supplement: Supplementary file 1 [file sb5c00629_si_001.pdf]

SUPPORTING INFORMATION

Slowpoke: An Automated Golden Gate Cloning Workflow for Opentrons OT-2 and Flex

Koray Malcı[1,2,a,*], Fankang Meng[1,2,a], Henri Galez[3], Alicia Franja Da Silva [3,4], Joaquin Caro-Astorga[1,2,5,6], Gregory Batt[3], Tom Ellis[1,2]

[1] Department of Bioengineering, Imperial College London, London, SW7 2AZ, UK

[2] Imperial Centre for Engineering Biology, Imperial College London, London, SW7 2AZ, UK

[3] Institut Pasteur, Inria, Université Paris Cité, Paris, France

[4] IFP Energies Nouvelles, Rueil-Malmaison, France

[5] London South Bank University, London, SE1 0AA, UK

[6] LSBU Bioscience and Bioengineering Research Centre, London, SE1 0AA, UK

[a] Contributed equally as the first author.

* To whom correspondence may be addressed. Email: k.malci@imperial.ac.uk

Table S1: Primers used in the study

Name	Sequence 5' - 3'	Purpose
S32	cgatatgggaaacaaaatattgcg	Forward primer binding to STK202 backbone
S33	ctagttgaacgcttccatcttc	Reverse primer binding to *GFPMut3b* CDS
P99	acaagcaacgatctccagga	Forward primer binding to ConLS in pWS064 / pIB0036
P95	gttccggctgtcttgcttag	Reverse primer binding to ConRE in pWS064
P11	cgtggcaattcgtcgca	Primer binding to ConLS in pWS064 / pIB0036
MFK818	ctgaactggccgataattgcagacg	ConS_Scar[S/1]-JC forward primer
MFK819	cgtctgcaattatcggccagttcag	ConS_Scar[S/1]-JC reverse primer
MFK820	ccaaaccagatgtcaacacagctac	Con1_Scar[1/E]&[1/2]-JC forward primer
MFK821	gtagctgtgttgacatctggtttgg	Con1_Scar[1/E]&[1/2]-JC reverse primer
MFK822	gatgcacacactggcttaagatgac	Con2_Scar[2/E]&[2/3]-JC forward primer
MFK823	gtcatcttaagccagtgtgtgcatc	Con2_Scar[2/E]&[2/3]-JC reverse primer
MFK824	gttctgtatgggcacagacaaccta	Con3_Scar[3/E]&[3/4]-JC forward primer
MFK825	taggttgtctgtgcccatacagaac	Con3_Scar[3/E]&[3/4]-JC reverse primer
MFK826	ggtagactacccatgagtcacaatgaac	Con4_Scar[4/E]&[4/5]-JC forward primer
MFK827	gttcattgtgactcatgggtagtctacc	Con4_Scar[4/E]&[4/5]-JC reverse primer
MFK828	aagtgaataaagctccacacagtcg	Con5_Scar[5/E]-JC forward primer
MFK829	cgactgtgtggagctttattcactt	Con5_Scar[5/E]-JC reverse primer
MFK830	agcagttacagagatgttacgaacc	ConE_Scar-JC forward primer
MFK831	ggttcgtaacatctctgtaactgct	ConE_Scar-JC reverse primer
MFK832	cagactggcaccgacaaagcaa	Leu5' Homology-JC forward primer
MFK833	tttgtgtggtgccctcctcctt	Leu3' Homology-JC reverse primer
MFK834	aggcaagacaagcaacgaaacgt	Ura5' Homology-JC forward primer
MFK835	tgttacttggttctggcgaggt	Ura3' Homology-JC reverse primer
MFK836	tgccctcaagaaacgaggctct	HO5' Homology-JC forward primer
MFK837	cctacgctcagggcactgtact	HO3' Homology-JC reverse primer

Table S2: High-throughput DNA Assemblies using 6 YTK-compatible Parts in Flex

Plasmid Name	Part Combinations used in the Assemblies	Number of White Colonies	Bands on Agarose Gel	Sequencing
pIB1739	pTDH3-spMF.endolysin-GFP.tTDH1-pIB0036	13	Correct	Correct
pIB1740	pTDH3-spSUC2.endolysin-GFP.tTDH1-pIB0036	10	Correct	Correct
pIB1741	pTDH3-spYap3~TA57.endolysin-GFP.tTDH1-pIB0036	15	Correct	Not tested
pIB1742	pTDH3-spOST1~MF.endolysin-GFP.tTDH1-pIB0036	8	Correct	Not tested
pIB1743	pTDH3-spFLO10.endolysin-GFP.tTDH1-pIB0036	0	No band	Not tested
pIB1744	pTDH3-spSWP1.endolysin-GFP.tTDH1-pIB0036	10	Correct	Not tested
pIB1745	pTDH3-spFET3.endolysin-GFP.tTDH1-pIB0036	18	Correct	Not tested
pIB1746	pTDH3-spMID2.endolysin-GFP.tTDH1-pIB0036	9	Correct	Not tested
pIB1747	pTDH3-spGAS5.endolysin-GFP.tTDH1-pIB0036	10	Correct	Not tested
pIB1748	pTDH3-spMFapp8.endolysin-GFP.tTDH1-pIB0036	13	Correct	Not tested
pIB1749	pTDH3-spMFappOPT.endolysin-GFP.tTDH1-pIB0036	10	Correct	Not tested
pIB1750	pTEF1-spMF.endolysin-GFP.tTDH1-pIB0036	17	Correct	Correct
pIB1751	pTEF1-spSUC2.endolysin-GFP.tTDH1-pIB0036	13	Correct	Correct
pIB1752	pTEF1-spYap3~TA57.endolysin-GFP.tTDH1-pIB0036	19	Correct	Not tested
pIB1753	pTEF1-spOST1~MF.endolysin-GFP.tTDH1-pIB0036	22	Correct	Not tested
pIB1754	pTEF1-spFLO10.endolysin-GFP.tTDH1-pIB0036	13	Correct	Not tested
pIB1755	pTEF1-spSWP1.endolysin-GFP.tTDH1-pIB0036	4	Correct	Not tested
pIB1756	pTEF1-spFET3.endolysin-GFP.tTDH1-pIB0036	8	Correct	Not tested
pIB1757	pTEF1-spMID2.endolysin-GFP.tTDH1-pIB0036	13	Correct	Not tested
pIB1758	pTEF1-spGAS5.endolysin-GFP.tTDH1-pIB0036	11	Correct	Not tested
pIB1759	pTEF1-spMFapp8.endolysin-GFP.tTDH1-pIB0036	16	Correct	Not tested
pIB1760	pTEF1-spMFappOPT.endolysin-GFP.tTDH1-pIB0036	50	Correct	Not tested
pIB1761	pALD6-spMF.endolysin-GFP.tTDH1-pIB0036	11	Correct	Correct
pIB1762	pALD6-spSUC2.endolysin-GFP.tTDH1-pIB0036	21	Correct	Correct
pIB1763	pALD6-spYap3~TA57.endolysin-GFP.tTDH1-pIB0036	11	Correct	Not tested
pIB1764	pALD6-spOST1~MF.endolysin-GFP.tTDH1-pIB0036	37	Correct	Not tested
pIB1765	pALD6-spFLO10.endolysin-GFP.tTDH1-pIB0036	22	Correct	Not tested
pIB1766	pALD6-spSWP1.endolysin-GFP.tTDH1-pIB0036	0	No band	Not tested
pIB1767	pALD6-spFET3.endolysin-GFP.tTDH1-pIB0036	22	Correct	Not tested
pIB1768	pALD6-spMID2.endolysin-GFP.tTDH1-pIB0036	0	No band	Not tested
pIB1769	pALD6-spGAS5.endolysin-GFP.tTDH1-pIB0036	22	Correct	Not tested
pIB1770	pALD6-spMFapp8.endolysin-GFP.tTDH1-pIB0036	0	No band	Not tested
pIB1771	pALD6-spMFappOPT.endolysin-GFP.tTDH1-pIB0036	18	Correct	Not tested
pIB1772	pTEF2-spMF.endolysin-GFP.tTDH1-pIB0036	20	Correct	Correct
pIB1773	pHHF1-spMF.endolysin-GFP.tTDH1-pIB0036	20	Correct	Correct
pIB1774	pHHF2-spMF.endolysin-GFP.tTDH1-pIB0036	14	Correct	Correct
pIB1775	pHTB2-spMF.endolysin-GFP.tTDH1-pIB0036	0	No band	Not tested
pIB1776	pLight-spMF.endolysin-GFP.tTDH1-pIB0036	22	Correct	Correct
pIB1777	pTEF2-spSUC2.endolysin-GFP.tTDH1-pIB0036	12	Correct	Correct
pIB1778	pHHF1-spSUC2.endolysin-GFP.tTDH1-pIB0036	17	Correct	Correct
pIB1779	pHHF2-spSUC2.endolysin-GFP.tTDH1-pIB0036	28	Correct	Correct
pIB1780	pHTB2-spSUC2.endolysin-GFP.tTDH1-pIB0036	11	Correct	Not tested

pIB1781	pLight-spSUC2.endolysin-GFP.tTDH1-pIB0036	21	Correct	Correct
pIB1782	pTEF2-spYap3~TA57.endolysin-GFP.tTDH1-pIB0036	10	Correct	Not tested
pIB1783	pHHF1-spYap3~TA57.endolysin-GFP.tTDH1-pIB0036	22	Correct	Not tested
pIB1784	pHHF2-spYap3~TA57.endolysin-GFP.tTDH1-pIB0036	11	Correct	Not tested
pIB1785	pHTB2-spYap3~TA57.endolysin-GFP.tTDH1-pIB0036	0	No band	Not tested
pIB1786	pLight-spYap3~TA57.endolysin-GFP.tTDH1-pIB0036	30	Correct	Not tested
pIB1787	pTEF2-spOST1~MF.endolysin-GFP.tTDH1-pIB0036	4	Correct	Not tested
pIB1788	pHHF1-spOST1~MF.endolysin-GFP.tTDH1-pIB0036	4	Correct	Not tested
pIB1789	pHHF2-spOST1~MF.endolysin-GFP.tTDH1-pIB0036	6	Correct	Not tested
pIB1790	pHTB2-spOST1~MF.endolysin-GFP.tTDH1-pIB0036	0	No band	Not tested
pIB1791	pLight-spOST1~MF.endolysin-GFP.tTDH1-pIB0036	30	Correct	Not tested
pIB1792	pLight-spFET3.scFv-alphaF.tTDH1-pWS064	30	Correct	Not tested
pIB1793	pLight-spGAS5.scFv-alphaF.tTDH1-pWS064	2	Correct	Correct
pIB1794	pLight-spMID2.scFv-alphaF.tTDH1-pWS064	13	Correct	Correct
pIB1795	pLight-spOST1~MF.scFv-alphaF.tTDH1-pWS064	8	Correct	Correct
pIB1796	pLight-spSUC2.scFv-alphaF.tTDH1-pWS064	10	Correct	Correct
pIB1797	pLight-spSWP1.scFv-alphaF.tTDH1-pWS064	1	Correct	Correct
pIB1798	pLight-spYap3~TA57.scFv-alphaF.tTDH1-pWS064	13	Correct	Correct
pIB1799	pLight-spMF.scFv-alphaF.tTDH1-pWS064	30	Correct	Correct
pIB1800	pLight-spFLO10.scFv-alphaF.tTDH1-pWS064	30	Correct	Correct

Table S3: Details of user time and machine time needed for large scale Flex workflow

Workflow	Tasks		User time (minutes)	Machine time (minutes)	Machine	Comments
Golden Gate	Dry lab setup	Construct 64 plasmid maps	20	0		Generated with InSillyClo.
		CSV file preparation	20	0		
		Script generation	10	0		
	Wet lab setup	Fixed toolkit preparation	45	0		
		6WP agar plate preparation	60	0		12 6-well plates prepared.
	Robot run	Mix buffer and plasmids	10	180	Flex	Users need to prepare the tube with buffer and water.
		Enzyme addition	10	30	Flex	Users need to prepare the tube with the enzymes.
		Golden Gate reaction	0	240	Thermocycler	Could be done overnight.
		Competent cell addition	60	60	Flex	Keep each tube on ice before use.
		Transformation	0	75	Thermocycler	
		Plating	60	60	Flex	Users need to be replace 6WP plates.
	Post processing	Growth	0	1440	Incubator	24h in the 37°C incubator.
		Colony count	30	0		
		Restrike on fresh medium	90	0		One colony per assembly is transferred.
		Growth	0	960	Incubator	Overnight (16h) in the 37°C incubator.
Colony PCR	Dry lab setup	CSV file preparation	20	0		
		Script generation	10	0		
	Wet lab setup	Colony template plate preparation	30	0		
	Robot run	Master mix preparation	10	10	Flex	Manual mixing if big volume.
		Reaction plate preparation	0	**60**	Flex	
		PCR reaction	0	180	Thermocycler	
	Post processing	Agarose Gel electrophoresis	90	120	Electrophoresis device	
		PCR band analysis	15	0		Theoretical bands simulated with InSillyClo.
Storage and purification		Liquid culture preparations	45	0		
		Growth	0	960	Shaking incubator	Overnight (16h) in the 37°C incubator.
		Strain storage	120	0		
		Plasmid purification	360	0		Done in 3 batches (approx. 2h)

Table S4: High-throughput Colony PCR Details

PCR Number	Plasmids	Primers1	Primer2	Expected Length (kb)
1	pMFK-754	MFK834	MFK821	2.1
2	pMFK-761	MFK834	MFK821	2.1
3	pMFK-765	MFK834	MFK821	2.1
4	pMFK-756	MFK834	MFK821	2.1
5	pMFK-759	MFK834	MFK821	2.1
6	pMFK-758	MFK834	MFK821	2.1
7	pMFK-757	MFK834	MFK821	2.1
8	pMFK-762	MFK834	MFK821	2.1
9	pMFK-760	MFK834	MFK821	2.1
10	pMFK-764	MFK834	MFK821	2.1
11	pMFK-763	MFK834	MFK821	2.1
12	pMFK-755	MFK834	MFK821	2.1
13	pMFK-834	MFK834	MFK821	2.2
14	pMFK-836	MFK834	MFK821	2.2
15	pMFK-837	MFK834	MFK821	2.2
16	pMFK-835	MFK834	MFK821	2.2
17	pMFK-833	MFK834	MFK821	2.2
18	pMFK-657	MFK834	MFK821	2.2
19	pMFK-665	MFK834	MFK821	2.2
20	pMFK-659	MFK834	MFK821	2.2
21	pMFK-667	MFK834	MFK821	2.2
22	pMFK-661	MFK834	MFK821	2.2
23	pMFK-663	MFK834	MFK821	2.2
24	pMFK-008	MFK834	MFK821	2.1
25	pMFK-059	MFK834	MFK821	2.1
26	pMFK-125	MFK834	MFK821	2.1
27	pMFK-095	MFK834	MFK821	2.1
28	pMFK-053	MFK834	MFK821	2.1
29	pMFK-050	MFK834	MFK821	2.1
30	pMFK-056	MFK834	MFK821	2.1
31	pMFK-104	MFK834	MFK821	2.1
32	pMFK-062	MFK834	MFK821	2.1
33	pMFK-101	MFK834	MFK821	2.1
34	pMFK-110	MFK834	MFK821	2.1
35	pMFK-107	MFK834	MFK821	2.1
36	pMFK-092	MFK834	MFK821	2.1
37	pMFK-648	MFK834	MFK821	2.1
38	pMFK-215	MFK834	MFK821	2.1
39	pMFK-971	MFK834	MFK821	2.1
40	pMFK-972	MFK834	MFK821	2.1
41	pMFK-089	MFK834	MFK821	2.1
42	pMFK-669	MFK834	MFK821	2.1
43	pMFK-649	MFK834	MFK821	2.1
44	pMFK-973	MFK834	MFK821	2.1
45	pMFK-975	MFK834	MFK821	2.1
46	pMFK-974	MFK834	MFK821	2.1
47	pMFK-293	MFK834	MFK821	2.2
48	pMFK-294	MFK834	MFK821	2.2
49	pMFK-295	MFK834	MFK821	2.2
50	pMFK-296	MFK834	MFK821	2.2
51	pMFK-297	MFK834	MFK821	2.2
52	pMFK-298	MFK834	MFK821	2.2
53	pMFK-299	MFK834	MFK821	2.2
54	pMFK-300	MFK834	MFK821	2.2
55	pMFK-301	MFK834	MFK821	2.2
56	pMFK-302	MFK834	MFK821	2.2
57	pMFK-303	MFK834	MFK821	2.2
58	pMFK-754	MFK820	MFK823	3.2
59	pMFK-761	MFK820	MFK823	3.2
60	pMFK-765	MFK820	MFK823	3.2
61	pMFK-756	MFK820	MFK823	3.2

62	pMFK-759	MFK820	MFK823	3.2
63	pMFK-758	MFK820	MFK823	3.2
64	pMFK-757	MFK820	MFK823	3.2
65	pMFK-762	MFK820	MFK823	3.2
66	pMFK-760	MFK820	MFK823	3.2
67	pMFK-764	MFK820	MFK823	3.2
68	pMFK-763	MFK820	MFK823	3.2
69	pMFK-755	MFK820	MFK823	3.2
70	pMFK-834	MFK820	MFK823	3.3
71	pMFK-836	MFK820	MFK823	3.3
72	pMFK-837	MFK820	MFK823	3.3
73	pMFK-835	MFK820	MFK823	3.3
74	pMFK-833	MFK820	MFK823	3.3
75	pMFK-657	MFK820	MFK823	3.3
76	pMFK-665	MFK820	MFK823	3.3
77	pMFK-659	MFK820	MFK823	3.3
78	pMFK-667	MFK820	MFK823	3.3
79	pMFK-661	MFK820	MFK823	3.3
80	pMFK-663	MFK820	MFK823	3.3
81	pMFK-008	MFK820	MFK823	3.2
82	pMFK-059	MFK820	MFK823	3.2
83	pMFK-125	MFK820	MFK823	3.2
84	pMFK-095	MFK820	MFK823	3.2
85	pMFK-053	MFK820	MFK823	3.2
86	pMFK-050	MFK820	MFK823	3.2
87	pMFK-056	MFK820	MFK823	3.2
88	pMFK-104	MFK820	MFK823	3.2
89	pMFK-062	MFK820	MFK823	3.2
90	pMFK-101	MFK820	MFK823	3.2
91	pMFK-110	MFK820	MFK823	3.2
92	pMFK-107	MFK820	MFK823	3.2
93	pMFK-092	MFK820	MFK823	3.2
94	pMFK-648	MFK820	MFK823	3.2
95	pMFK-215	MFK820	MFK823	3.2
96	pMFK-971	MFK820	MFK823	3.2
97	pMFK-972	MFK820	MFK823	3.2
98	pMFK-089	MFK820	MFK823	3.2
99	pMFK-669	MFK820	MFK823	3.2
100	pMFK-649	MFK820	MFK823	3.2
101	pMFK-973	MFK820	MFK823	3.2
102	pMFK-975	MFK820	MFK823	3.2
103	pMFK-974	MFK820	MFK823	3.2
104	pMFK-293	MFK820	MFK823	3.3
105	pMFK-294	MFK820	MFK823	3.3
106	pMFK-295	MFK820	MFK823	3.3
107	pMFK-296	MFK820	MFK823	3.3
108	pMFK-297	MFK820	MFK823	3.3
109	pMFK-298	MFK820	MFK823	3.3
110	pMFK-299	MFK820	MFK823	3.3
111	pMFK-300	MFK820	MFK823	3.3
112	pMFK-301	MFK820	MFK823	3.3
113	pMFK-302	MFK820	MFK823	3.3
114	pMFK-303	MFK820	MFK823	3.3
115	pMFK-754	MFK822	MFK831	1.3
116	pMFK-761	MFK822	MFK831	1.3
117	pMFK-765	MFK822	MFK831	1.3
118	pMFK-756	MFK822	MFK831	1.3
119	pMFK-759	MFK822	MFK831	1.3
120	pMFK-758	MFK822	MFK831	1.3
121	pMFK-757	MFK822	MFK831	1.3
122	pMFK-762	MFK822	MFK831	1.3
123	pMFK-760	MFK822	MFK831	1.3
124	pMFK-764	MFK822	MFK831	1.3
125	pMFK-763	MFK822	MFK831	1.3

126	pMFK-755	MFK822	MFK831	1.3
127	pMFK-834	MFK822	MFK831	1.4
128	pMFK-836	MFK822	MFK831	1.4
129	pMFK-837	MFK822	MFK831	1.4
130	pMFK-835	MFK822	MFK831	1.4
131	pMFK-833	MFK822	MFK831	1.4
132	pMFK-657	MFK822	MFK831	1.4
133	pMFK-665	MFK822	MFK831	1.4
134	pMFK-659	MFK822	MFK831	1.4
135	pMFK-667	MFK822	MFK831	1.4
136	pMFK-661	MFK822	MFK831	1.4
137	pMFK-663	MFK822	MFK831	1.4
138	pMFK-008	MFK822	MFK831	1.3
139	pMFK-059	MFK822	MFK831	1.3
140	pMFK-125	MFK822	MFK831	1.3
141	pMFK-095	MFK822	MFK831	1.3
142	pMFK-053	MFK822	MFK831	1.3
143	pMFK-050	MFK822	MFK831	1.3
144	pMFK-056	MFK822	MFK831	1.3
145	pMFK-104	MFK822	MFK831	1.3
146	pMFK-062	MFK822	MFK831	1.3
147	pMFK-101	MFK822	MFK831	1.3
148	pMFK-110	MFK822	MFK831	1.3
149	pMFK-107	MFK822	MFK831	1.3
150	pMFK-092	MFK822	MFK831	1.3
151	pMFK-648	MFK822	MFK831	1.3
152	pMFK-215	MFK822	MFK831	1.3
153	pMFK-971	MFK822	MFK831	1.3
154	pMFK-972	MFK822	MFK831	1.3
155	pMFK-089	MFK822	MFK831	1.3
156	pMFK-669	MFK822	MFK831	1.3
157	pMFK-649	MFK822	MFK831	1.3
158	pMFK-973	MFK822	MFK831	1.3
159	pMFK-975	MFK822	MFK831	1.3
160	pMFK-974	MFK822	MFK831	1.3
161	pMFK-293	MFK822	MFK831	1.4
162	pMFK-294	MFK822	MFK831	1.4
163	pMFK-295	MFK822	MFK831	1.4
164	pMFK-296	MFK822	MFK831	1.4
165	pMFK-297	MFK822	MFK831	1.4
166	pMFK-298	MFK822	MFK831	1.4
167	pMFK-299	MFK822	MFK831	1.4
168	pMFK-300	MFK822	MFK831	1.4
169	pMFK-301	MFK822	MFK831	1.4
170	pMFK-302	MFK822	MFK831	1.4
171	pMFK-303	MFK822	MFK831	1.4
172	pMFK-041	MFK824	MFK831	3.0
173	pMFK-044	MFK824	MFK831	3.0
174	pMFK-083	MFK824	MFK831	3.0
175	pMFK-112	MFK824	MFK831	3.0
176	pMFK-114	MFK824	MFK831	3.0
177	pMFK-116	MFK824	MFK831	3.0
178	pMFK-118	MFK824	MFK831	3.0
179	pMFK-122	MFK824	MFK831	3.0
180	pMFK-226	MFK824	MFK831	3.0
181	pMFK-227	MFK824	MFK831	3.0
182	pMFK-228	MFK824	MFK831	3.0
183	pMFK-229	MFK824	MFK831	3.0
184	pMFK-230	MFK824	MFK831	3.0
185	pMFK-619	MFK824	MFK831	3.0
186	pMFK-620	MFK824	MFK831	3.0
187	pMFK-621	MFK824	MFK831	3.0
188	pMFK-622	MFK824	MFK831	3.0
189	pMFK-624	MFK824	MFK831	3.0

190	pMFK-909	MFK411	MFK708	2.0
191	pMFK-910	MFK411	MFK708	2.0
192	pMFK-911	MFK411	MFK708	2.0
193	pMFK-912	MFK411	MFK708	2.0
194	pMFK-913	MFK411	MFK708	2.0
195	pMFK-914	MFK411	MFK708	2.0
196	pMFK-915	MFK411	MFK708	2.0
197	pMFK-916	MFK411	MFK708	2.0
198	pMFK-917	MFK411	MFK708	2.0
199	pMFK-918	MFK411	MFK708	2.0
200	pMFK-919	MFK411	MFK708	2.0
201	pMFK-920	MFK411	MFK708	2.0
202	pMFK-921	MFK411	MFK708	2.0
203	pMFK-922	MFK411	MFK708	2.0
204	pMFK-923	MFK411	MFK708	2.0
205	pMFK-924	MFK411	MFK708	2.0
206	pMFK-925	MFK411	MFK708	2.0
207	pMFK-926	MFK411	MFK708	2.0
208	pMFK-927	MFK411	MFK708	2.0
209	pMFK-928	MFK411	MFK708	2.0
210	pMFK-929	MFK411	MFK708	2.0
211	pMFK-930	MFK411	MFK708	2.0
212	pMFK-931	MFK411	MFK708	2.0
213	pMFK-932	MFK411	MFK708	2.0
214	pMFK-235	MFK834	MFK821	2.5
215	pMFK-044	MFK820	MFK823	2.1
216	pMFK-083	MFK820	MFK823	2.1
217	pMFK-114	MFK820	MFK823	2.1
218	pMFK-118	MFK820	MFK823	2.1
219	pMFK-141	MFK820	MFK823	3.0
220	pMFK-143	MFK820	MFK823	3.0
221	pMFK-624	MFK822	MFK825	2.5
222	pMFK-950	MFK822	MFK831	1.5
223	pMFK-951	MFK822	MFK831	1.5
224	pMFK-920	MFK411	MFK708	1.9
225	pMFK-932	MFK411	MFK708	1.9
226	pMFK-1131	MFK411	MFK708	1.9
227	pMFK-1132	MFK411	MFK708	1.9
228	pMFK-1135	MFK834	MFK821	2.1
229	pMFK-1141	MFK834	MFK821	2.1
230	pMFK-1135	MFK820	MFK823	1.2
231	pMFK-1141	MFK820	MFK823	1.2
232	pMFK-1135	MFK822	MFK831	1.5
233	pMFK-1141	MFK822	MFK831	1.5
234	pMFK-235	MFK820	MFK823	3.1
235	pMFK-235	MFK822	MFK831	1.2
236	pMFK-624	MFK824	MFK831	2.9

Table S5: Volume Details used in Protocols for Flex and OT-2 Platforms

Protocol / Component	Flex	OT-2	Notes
**Golden Gate**			
Total Reaction Volume	12 μL	10 μL	Flex uses a larger volume and supports N=6 parts by default.
Enzyme (μL)	1.2 μL	≈1.0 μL	
Buffer (μL)	1.2 μL	≈1.0 μL	
Water/Buffer Mix (μL)	4.8 μL (calculated: 12−1.2−6×1)	Variable: (8−N) μL	N is the number of parts. Flex protocol specifies N=6 by default, resulting in 4.8μL water/buffer.
DNA Input Parts (per part)	1.0 μL	1 μL	Number of parts (N) is 6 in the Flex protocol, 4 in the OT-2.
Competent Cells for Transformation	50 μL	50μL	Added after GG assembly.
**Colony PCR**			
Total Reaction Volume	15 μL	10 μL	Flex uses a larger total volume.
**Reaction Mix**	The total volume is 13 μL (Flex) or 9 μL (OT-2), with the remaining volume being the colony template.		
Water	2.5 μL	≈3 μL	
Enzyme/Buffer Mix	7.5 μL	5.0 μL	
Primer Forward	1.5 μL	1.0 μL	
Primer Reverse	1.5 μL	1.0 μL	
Colony Template (DNA)	2.0 μL	1 μL	Transferred separately.
**Plating**			
Volume Plated per Deposition Point	2.5μL	4.5μL	This is the volume dispensed per 'dot' on the agar plate.
Number of Deposition Points	13 points	13 points	
Total Volume Plated per Reaction	32.5 μL (13×2.5 μL)	58.5μL (13×4.5 μL)	The total liquid volume distributed from the reaction well onto the agar plate.

Table S6: Medium MC components

Medium MC (50 mL)	Volume	10x MC Master Mix in 50 mL
**10x MC Master Mix**	5 mL	5.356 g K_2HPO_4
**H_2O**	44 mL	2.62 g KH_2PO_4
**1M $MgSO_4$**	166 μL	10 g D-glucose
**Tryptophane (10 mg/ml)**	250 μL	0.5 g Casamino acids
**Phenylalanine (10 mg/ml)**	250 μL	1 g L-Glutamine (monopotasium salt)
		5 ml Ferric ammonium citrate (22 mg/mL)

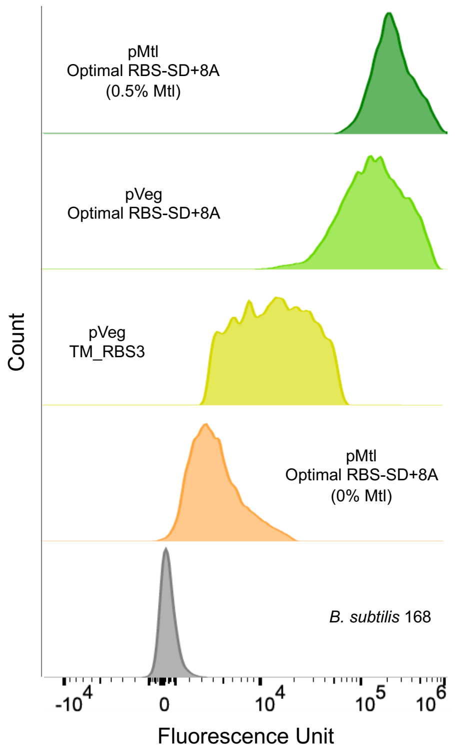

Figure S1: GFP expression levels measured by flow cytometry from different STK promoter–RBS combinations.

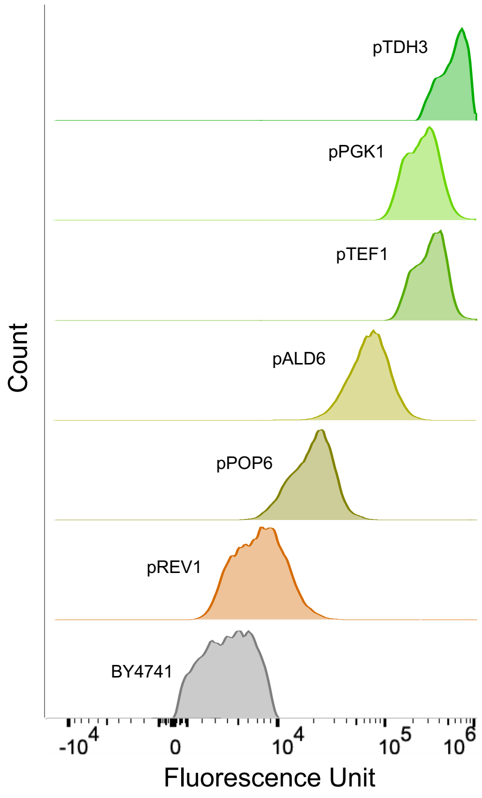

Figure S2: GFP expression levels measured by flow cytometry from different YTK promoters.

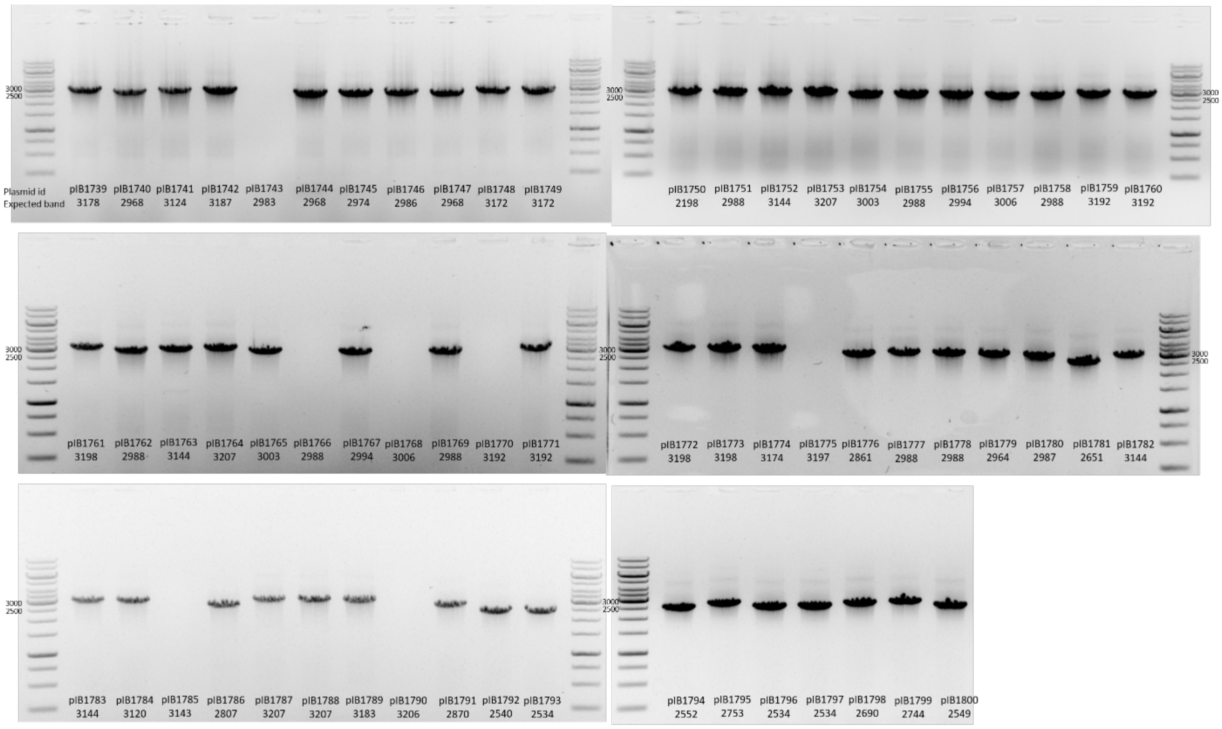

Figure S3: Colony PCR results for the large-scale assemblies performed in the Flex platform. 55 out of 62 assemblies showed expected band sizes. The remaining seven assemblies were likely to be toxic to E.coli, this was most probably because of the endolysin gene.

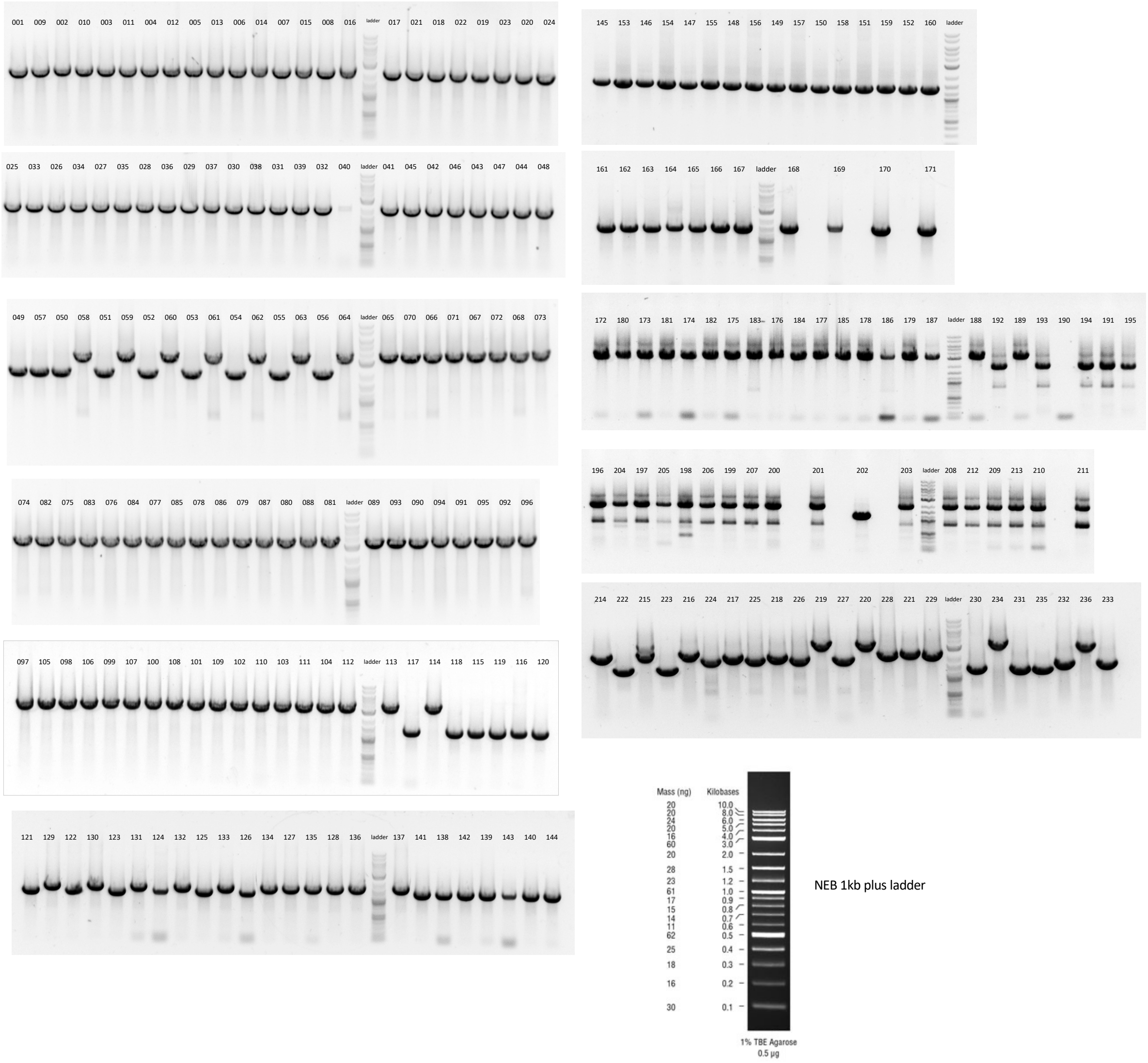

Figure S4: High-throughput colony PCR result performed by Opentrons OT-2 using three thermocyclers (one thermocycler module in OT-2, two additional bench-top thermocyclers). Gel images of 236 reactions out of 288 reactions are shown in the figure. Except for reaction 190, all bands had the expected sizes as shown in Table S2. The samples are in order in even-numbered or odd-numbered wells as they were loaded using a 12-channel multichannel pipette.

--- GUIDANCE ON SLOWPOKE ---

A detailed methodology for implementing Slowpoke in any standard biology lab is provided below.

Software:

- Opentrons App
- Python 3

Equipment and Consumables:

- OT-2 Basic Module or Flex Basic Module
- For OT-2: P20 Single-Channel Pipette (GEN2) or P10 Single-Channel Pipette (GEN1)
- For OT-2: P300 Single-Channel Pipette (GEN2) or P300 Single-Channel Pipette (GEN1)
- For Flex: Flex 1-Channel Pipette (1–50 µL)
- Thermocycler Module
- Temperature Module (2 if no Thermocycler Module)
- 24-well Aluminium Block
- 96-well Aluminium Block
- 24-well Tube Rack
- 6-Well Plate
- For OT-2: Tip Rack - 20 µL
- For OT-2: Tip Rack - 300 µL
- For Flex: Tip Rack - 50 µL
- PCR 96-Well Plate - 200 µL
- Adhesive PCR Plate Seals
- Reagents:
  - Type IIS Restriction Enzymes
  - T4 Ligase
  - ddH2O
  - PCR Master Mix (green master mixes such as the GoTaq Green Master Mix used for OT-2 or the Dreamtaq Master Mix used for the Flex)
- Competent *E. coli* cells
- LB agar with corresponding antibiotics

Golden Gate Cloning Protocol in OT-2

1. Get the necessary folders/files from https://github.com/Tom-Ellis-Lab/Slowpoke/tree/main/Cloning .
2. Open a command line (Terminal in Mac), set the working directory to the "moclo_transformation" folder
3. Run "**python3 generator_OT2_for_cloning_protocol.py**".
4. Select the three .csv files (fixed_toolkit_map, custom_parts_map, combination-to-make), the cloning workflow Python file and an output folder.
5. Outputs: "protocol_for_cloning.py" and "agar_plate.csv" will be saved in the output folder.
6. Upload "protocol_for_cloning.py" to Opentrons App under the "Protocols" tab.

7. In the Opentrons App, follow calibration instructions and place the following labware onto the OT-2 deck (Figure S5):

    7.1. Temperature module with an Opentrons 24-well aluminium block

    7.2. 1.5 mL tubes with water, Golden Gate mix (a Type IIS restriction enzyme, T4 ligase, T4 ligase buffer, water) on the 24-well aluminium block. The total Golden Gate mix volume should be adjusted according to the number of parts used in each reaction and the total number of reactions prepared. In a standard 10 µL Golden Gate reaction, 0.5 µL (≈ 50 fmol) of each DNA part is added. To prepare a master mix, we recommend calculating the total required volume as

    (Total reaction number + 2) × [10 µL – (part number × 0.5 µL)],

    where two extra reactions are included to compensate for pipetting losses.

    7.3. A plate for *fixed_input_DNA_map*

    7.4. A plate for *customised_input_DNA_map*

    7.5. One 10 µL and two 300 µL tip racks

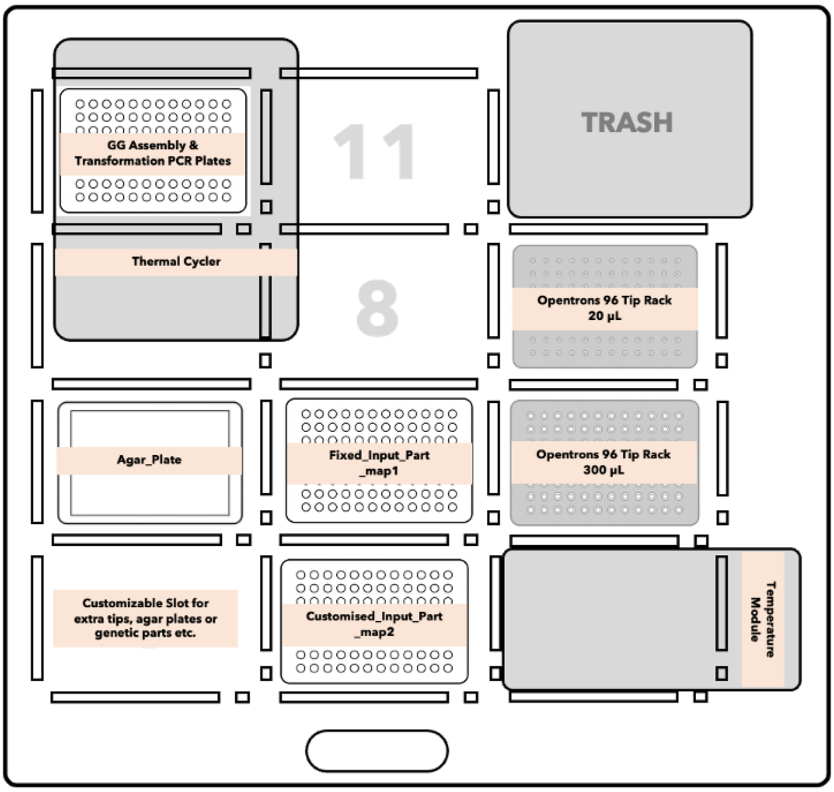

Figure S5: The OT-2 deck layout for the automated Golden Gate cloning

8. Run the protocol.
9. The robot pauses when the transfers are completed. Seal the reaction plate on the thermocycler module and resume in the Opentrons App for DNA assembly.
10. The robot pauses when the Golden Gate program is completed. Hold the temperature module at 4 °C. Place the competent cell tube onto the 24-well aluminium block. The protocol is configured to use **50 µL of competent *E. coli*** cells per reaction, which is generally sufficient. If transformation efficiency is low, the volume of competent cells may be increased as needed. The total transformation mix should not exceed **100 µL**, due to the capacity limitations of the thermocycler modules. All competent cells can be

prepared in a single tube, as the pipette tip is automatically changed between each transformation step.

11. Remove the adhesive film and resume in the Opentrons App to proceed with adding competent cells into the reaction plate.
12. The robot pauses when the steps are completed. Seal the reaction plate and resume the protocol in the Opentrons App for heat shock.
13. The robot pauses when the heat shock is completed. Place LB agar plate on deck. Resume for plating.
14. If more plates are needed, pause after one round, change plates, and resume plating on a new LB agar plate.
15. Remove the agar plate and incubate at 37 °C overnight.

Golden Gate Cloning Protocol in Flex

This protocol can be used for low-throughput or high-throughput Golden Gate based cloning as presented in this study.

1. Get the necessary folders/files from https://github.com/Tom-Ellis-Lab/Slowpoke/tree/main/Cloning.
2. Open a command line (Terminal in Mac), set the working directory to the "moclo_transformation" folder
3. Run "**python3 generator_Flex_for_cloning_protocol.py**".
4. Select the three .csv files (fixed_toolkit_map, custom_parts_map, combination-to-make), the cloning workflow Python file and an output folder.
5. Outputs: "protocol_for_cloning.py" and "agar_plate.csv" will be saved in the output folder.
6. Upload "protocol_for_cloning.py" to Opentrons App under the "Protocols" tab.
7. In the Opentrons App, follow calibration instructions and place the following labware onto the Flex deck (Figure S6):
    - 7.1. Reagent temperature module + an Opentrons 24-well aluminium block on D3 deck position
        - i. 1.5 mL tube with water and buffer in A1 of the Reagent temperature module
        - ii. 1.5 mL tube with enzyme mix in B1 of the Reagent temperature module (can be placed at the last moment with a 8 µl dead volume)
    - 7.2. Temperature module + an Opentrons 96-well aluminum block + 96-well plate on A1 deck position
    - 7.3. The 96-well plate for *fixed_input_DNA_map* on C2 deck position
    - 7.4. 50 µL tip racks on A2, B1, B2, B3, C3, D1 and D2 deck positions.
8. The robot starts by adding water and buffer to the reaction plate for all reactions. Then, the input plasmids are added. Next, the protocol pauses to let you add the enzyme tube. Once enzymes are added, the protocol pauses again so you can seal the plate and transfer it to the thermocycler.

Thermocycler program for Golden Gate: (37°C 2 min + 16°C 5 min) x 25 → 60 °C 5 min → 4 °C

9. Place the reaction plate on the temperature module in A1 deck position and put the competent cell tube in the D1, D2, D3 and D4 positions of the temperature module in D3 deck position. Tubes of competent cell were kept on ice and placed on the deck just before their use.
10. The robot transfers cells to each Golden Gate reaction and mix gently by pipetting. The protocol pauses to let you take the plate to the thermocycler for the transformation.

    Thermocycler program for Transformation: 4°C 10 min → 42°C 30 sec → 4°C 2 min → 37°C 1h

11. Place the reaction plate in A1 deck position and LB agar plate on C1 deck position. Resume for plating.
12. If more plates are needed, pause after one round, change plates, and resume plating on a new LB agar plate.
13. Remove the agar plate and incubate at 37 °C overnight.

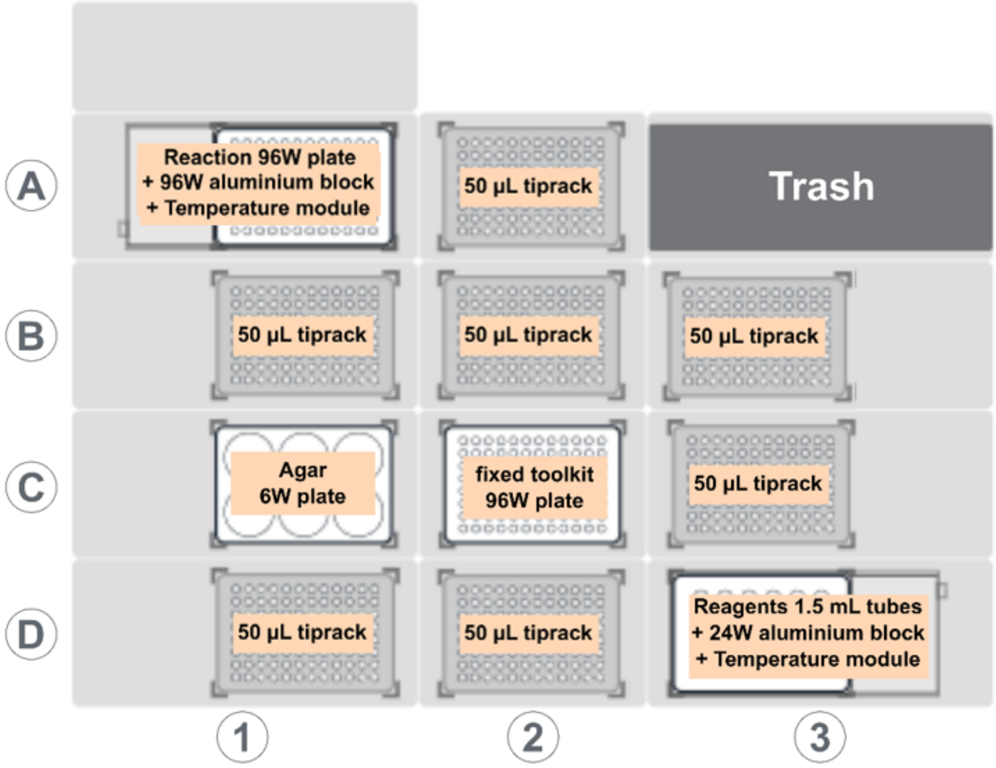

Figure S6: The Flex deck layout for the automated Golden Gate cloning

Notes for users:

- In the present study, we used only a single fixed-input DNA plate on the Flex due to deck-space constraints during large assembly experiments. However, the Flex workflow fully supports the use of a second DNA input plate for custom parts, following the same structure as in the OT-2 workflow. Users who wish to include a **custom_DNA_map** can simply enable the commented line in the script and place the corresponding plate at deck position D2.
- 10 µL of dead volume can be used for heavily used input plasmids, for instance, backbones
- For assemblies with many parts (>6), the amount of water & buffer added into the reactions wells at first will be low. Pipetting small volumes into an empty well can be challenging. We advise checking the correct dispense volume and tuning reaction parameters, for instance, increasing the total volume if needed.
- Workflows with either more assemblies or more parts per assembly will need more tip racks. Additional deck positions can be added with an expansion set from Opentrons, or the script can be modified to the tip racks.
- In large workflows, competent cells stay on the deck for some time. Even if they are kept at 4°C, transformation might be affected, especially for assembly numbers larger than those presented in this study. This could be achieved by manually dispensing competent cells, which is fast with an electronic pipette, or by placing cells in a 96-well plate and using the 8-channel Opentrons pipette to transfer them to the reaction plate. Additionally, LB could be added manually or via the robot during the recovery phase.

Colony PCR Protocol in OT-2

1. Get the necessary folders/files from https://github.com/Tom-Ellis-Lab/Slowpoke/tree/main/Colony_PCR.
2. Open a command line (Terminal on Mac) and set the working directory to the "Colony_PCR" folder.
3. Run **"python3 generator_for_colony_PCR_protocol.py".**
4. Select the three CSV files (pcr_deck_map, colony_template_map, pcr_recipe_to_make), the colony PCR workflow Python file and an output folder.
5. The "colony_PCR_protocol_date.py" file is automatically saved in the output folder.
6. Upload colony_PCR_protocol_date.py to Opentrons App under the "Protocols" tab.
7. In the Opentrons App, follow calibration instructions and place the following labware onto the OT-2 deck (Figure S7):
    - 7.1. A well plate for the *colony_template_map.csv*
    - 7.2. 1.5 mL tubes with PCR mix, water and primers on the 24-well tube rack
    - 7.3. Empty 1.5 mL tubes onto the 24-well tube rack
    - 7.4. Opentrons 10 µL and two 300 µL tip racks

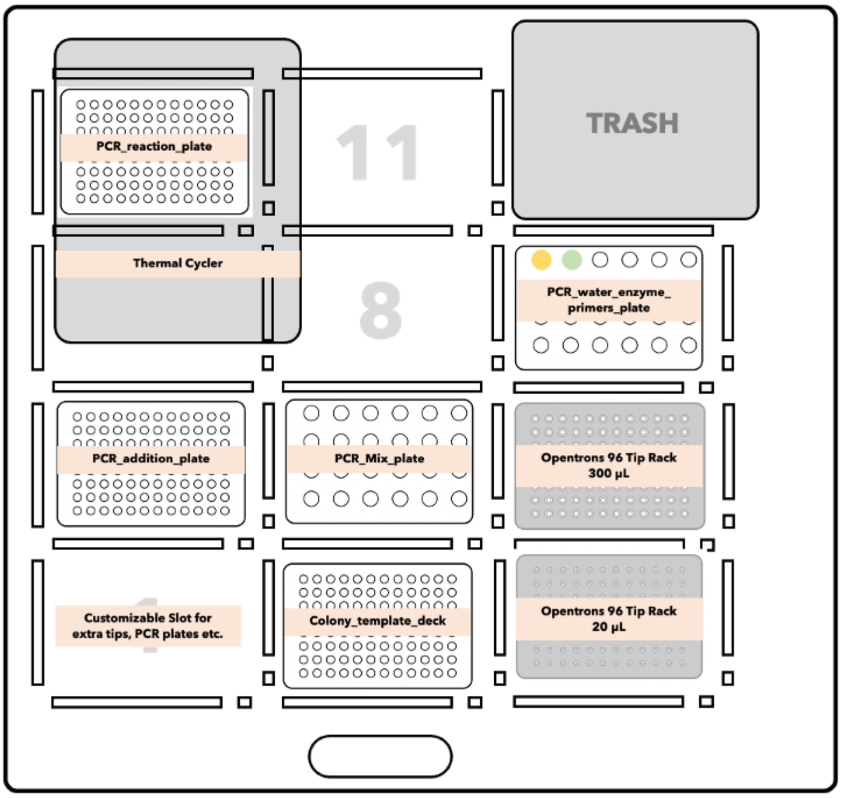

Figure S7: The OT-2 deck layout for the automated Colony PCR

8. Run the protocol.
9. The robot pauses when the transfers are completed. Seal the reaction plate and resume the protocol in the Opentrons App for the colony PCR program corresponding to the DNA polymerase or master mix used.
10. The robot pauses when colony PCR programs end. PCR products are ready for gel imaging and further analysis.

Colony PCR Protocol in Flex

1. Open a command line (Terminal on Mac) and set the working directory to your PCR colony folder.
2. Run **"python3 generator_for_colony_PCR_protocol.py".**
3. Select the three CSV files (pcr_deck_map, colony_template_map, pcr_recipe_to_make), the colony PCR workflow Python file and an output folder.
4. The "colony_PCR_protocol_date.py" file is automatically saved in the output folder.
5. Upload colony_PCR_protocol_date.py to Opentrons App under the "Protocols" tab.
6. In the Opentrons App, follow calibration instructions and place the following labware onto the Flex deck (Figure S8):
    a. Temperature module + an Opentrons 24-well aluminum block on D3 deck position with reagents in 1.5 µL tubes (primers, water, buffer and enzyme), mapping the pcr_deck_map
    b. Temperature module + an Opentrons 96-well aluminum block + 96-well plate on A1 deck positon

c. Empty 1.5mL tubes onto the 24-well tube rack on D2 deck position
d. 50 µL tip racks on C3, B3 and A2 deck positions
e. An additional 96-well plate on D1 deck position
f. The 96-well plate containing resuspended colonies on B2 deck position

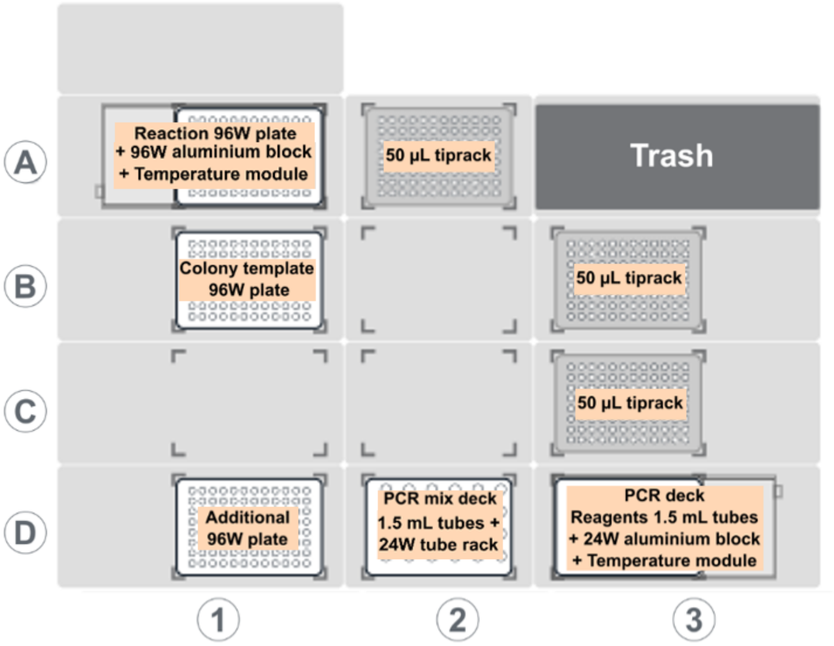

Figure S8: The Flex deck layout for the automated Colony PCR

7. Run the protocol.
8. The robot pauses when master mixes are done to give the opportunity to mix it manually if needed (easily removed from the protocol if unwanted).
9. The robot dispenses the master mixes and transfer 2µL of the colony template to the reaction well.
10. The robot pauses when the transfers are completed. Seal the reaction plate and transfer it to the thermocycler with appropriate PCR program.

Notes for users:

- The use of a 1000 µL pipette from Opentrons can save time for master mix preparation and automate mixing of large volumes
- Master mix volume is multiplied by 120% to ensure enough dead volume, so reagent volumes should be prepared accordingly

User Guide for Slowpoke GUI

1. Open the Slowpoke GUI via https://slowpoke.streamlit.app
2. Download the template files and the Python program on the left bar (Figure S9).
3. A detailed ReadMe file is also available among the template files.
4. Edit the template files depending on the specific entries.
5. Upload the edited template files and the Python program to the “Input Files” section under MoClo – Golden Gate or Colony PCR tabs.

6. Click the “Generate Protocol” button under the “Protocol Generation” section. This will make the protocol file and the final plate map.
7. Upload the produced protocol to the Opentrons App and run the protocol.

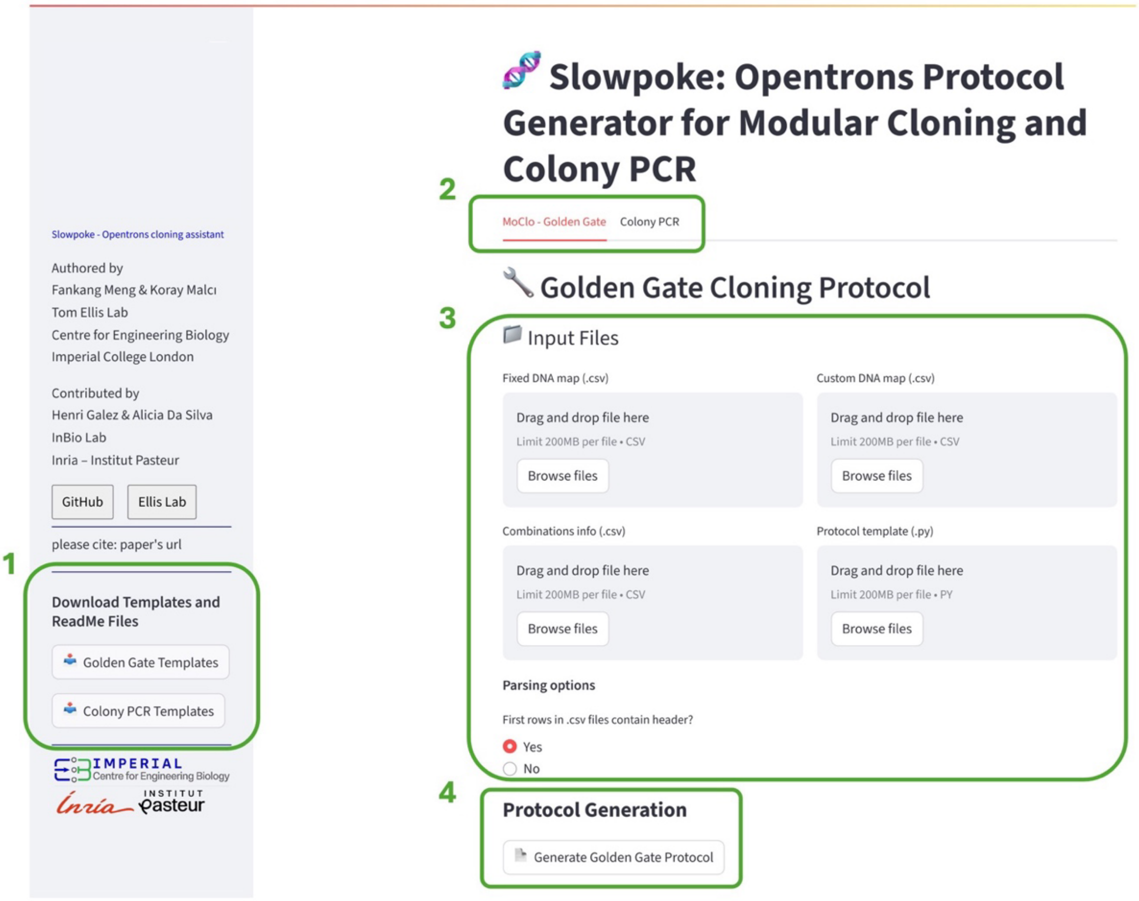

Figure S9: The user-friendly graphical user interface (GUI) of Slowpoke. (1) Template .csv and ReadMe files can be downloaded, (2) the desired protocol is selected, (3) user-specified input files are uploaded, and (4) the protocol is generated with an instant preview.

Using the Template Files in the Slowpoke GUI:

- The provided `.csv` templates are pre-filled with example entries to show the correct format.
- Replace the example entries with your own parts/reagents before running the app.
- Headers are recommended for clarity (default setting). The app also supports `.csv` files without headers. Figure S10 shows the headers of two sample input files.

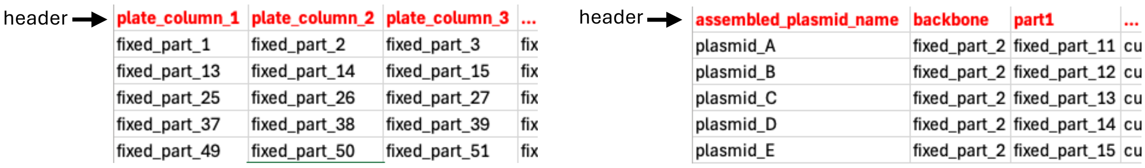

plate_column_1	plate_column_2	plate_column_3	...
fixed_part_1	fixed_part_2	fixed_part_3	fix
fixed_part_13	fixed_part_14	fixed_part_15	fix
fixed_part_25	fixed_part_26	fixed_part_27	fix
fixed_part_37	fixed_part_38	fixed_part_39	fix
fixed_part_49	fixed_part_50	fixed_part_51	fix

assembled_plasmid_name	backbone	part1	...
plasmid_A	fixed_part_2	fixed_part_11	cu
plasmid_B	fixed_part_2	fixed_part_12	cu
plasmid_C	fixed_part_2	fixed_part_13	cu
plasmid_D	fixed_part_2	fixed_part_14	cu
plasmid_E	fixed_part_2	fixed_part_15	cu

Figure S10: Headers in the input files were placed to minimise the confusion due to the notation difference between the spreadsheet and the physical plates.

Header Selection in the “Parsing Options”:

- If your `.csv` file includes headers, select ‘Yes’.

- If your `.csv` file does not include headers, select ‘No’.

Important Rules:

- Case-sensitive entries: Names in your source plate (e.g., `fixed_toolkit_map`) must exactly match the names in your destination plate (e.g., `combination_to_make`).
- For colony PCR, template values must exactly match entries in your colony template plate map.

Before Downloading Your Protocol:

- Always review the final assembly preview in the app to confirm everything is correct.

Important Notes

- Please ensure that the `.csv` files containing plate maps are in correspondence with the physical arrangement of a microplate. It's important to adhere to the standard row order (A-H) and column sequence (1-12) for a 96-well plate.
- In the combinations-to-make.csv file, the first column designates arbitrary names for the assembled plasmids, while the subsequent columns specify the combinations derived from the fixed_toolkit_map.csv and custom_parts_map.csv files.
- The two-plate layout, containing the *fixed_toolkit_map.csv* and *custom_parts_map.csv* files, was designed to simplify the combination of standardised and user-specific DNA parts. In practice, laboratories often create a working copy of standardised toolkits obtained from Addgene and extend them with their own custom parts. Nevertheless, users are free to select and combine parts from either or both plates when generating protocols.
- The online GUI supports .csv files with or without headers, which can be specified under the *parsing* options. Headers are included to clarify the mapping between the .csv structure and the physical well-plate layout, since there is a mismatch between spreadsheet notation and plate notation. In spreadsheet files, columns are labelled alphabetically (A–H or A–Z) and rows numerically (1–12), whereas in well plates this order is reversed. To minimise confusion, the provided templates include explicit headers such as “plate_column_1.”
- In the template files, example entries such as fixed_part_1 indicate placeholder plasmids corresponding to the first standardised part in the toolkit (for instance, *YTK001* in the Yeast Toolkit). Users should replace these placeholders with the actual part names when editing the templates.
- Detailed instructions on using the GUI, including the definition of each column header and example input files for both the cloning and colony PCR workflows, are provided in the online README documentation.

Troubleshooting

Problem	Likely cause	Recommended action
Protocol fails; missing wells	Part IDs in combinations don not exist in maps	Correct typos; double check case-sensitive matches or spaces
Tips run out mid-run	Insufficient tips or tip racks	Calculate tip usage, provide sufficient racks
Tip crashes	Wrong labware or wrong calibration	Double-check labware and their definitions, re-calibrate the robot, check the deck layout.
Low/no colonies	Inactive enzymes, wrong cycling, inefficient competent cells, need of recovery	Keep enzymes at 4 °C, double-check the Gelden Gate cycles, confirm the enzyme activity and competent cells' efficiency with a manual test. After heat-shock perform a proper recovery in LB or SOC media.
Backbone recircularization	Incomplete digestion, high backbone mass	Adjust insert:backbone fmol, verify the restriction enzymes with manual tests, use dropout markers.
Low transformation	> 100 µL in wells, timing off	Cap total transformation volume at 100 µL, use 50 µL/reaction (increase if needed), adjust the timing if needed.
No spread, breaking/tearing agar media	Cold/condensed plates, incorrect Z-height calibration/definition, too thick agar media	Equilibrate plates, brief drying, adjust Z-height accordingly, standardise the agar media thickness with a fixed volume in 6-well plates, optional manual spread per policy,
False negatives in colony PCR	Too much biomass, short extensions	Very small colony input, increase extension for long amplicons, match Tm.
Cross-contamination in colony PCR	Tip reuse; aggressive mixing	Force new tips, reduce mixing speed/cycles, seal plates before PCR.
